# Quantifying Policy Options for Reducing Future Coronary Heart Disease Mortality in England: A Modelling Study

**DOI:** 10.1371/journal.pone.0069935

**Published:** 2013-07-25

**Authors:** Shaun Scholes, Madhavi Bajekal, Paul Norman, Martin O’Flaherty, Nathaniel Hawkins, Mika Kivimäki, Simon Capewell, Rosalind Raine

**Affiliations:** 1 Department of Applied Health Research, University College London, London, United Kingdom; 2 School of Geography, University of Leeds, Leeds, United Kingdom; 3 Institute of Cardiovascular Medicine & Science, Liverpool Heart and Chest Hospital, University of Liverpool, Liverpool, United Kingdom; 4 Institute of Psychology, Health and Society, University of Liverpool, Liverpool, United Kingdom; 5 Department of Epidemiology and Public Health, University College London, London, United Kingdom; Universidad Peruana de Ciencias Aplicadas (UPC), Peru

## Abstract

**Aims:**

To estimate the number of coronary heart disease (CHD) deaths potentially preventable in England in 2020 comparing four risk factor change scenarios.

**Methods and Results:**

Using 2007 as baseline, the IMPACT_SEC_ model was extended to estimate the potential number of CHD deaths preventable in England in 2020 by age, gender and Index of Multiple Deprivation 2007 quintiles given four risk factor change scenarios: (a) assuming recent trends will continue; (b) assuming optimal but feasible levels already achieved elsewhere; (c) an intermediate point, halfway between current and optimal levels; and (d) assuming plateauing or worsening levels, the worst case scenario. These four scenarios were compared to the baseline scenario with both risk factors and CHD mortality rates remaining at 2007 levels. This would result in approximately 97,000 CHD deaths in 2020. Assuming recent trends will continue would avert approximately 22,640 deaths (*95% uncertainty interval: 20,390-24,980*). There would be some 39,720 (37,120-41,900) fewer deaths in 2020 with optimal risk factor levels and 22,330 fewer (*19,850-24,300*) in the intermediate scenario. In the worst case scenario, 16,170 additional deaths (*13,880-18,420*) would occur. If optimal risk factor levels were achieved, the gap in CHD rates between the most and least deprived areas would halve with falls in systolic blood pressure, physical inactivity and total cholesterol providing the largest contributions to mortality gains.

**Conclusions:**

CHD mortality reductions of up to 45%, accompanied by significant reductions in area deprivation mortality disparities, would be possible by implementing optimal preventive policies.

## Introduction

Recent UK declines in the rate of coronary heart disease (CHD) mortality have been impressive. The wider use of treatments among people with CHD explains around half the recent fall [1]. Just over one third is explained by population trends in major cardiovascular risk factors, namely falls in blood pressure, cholesterol, and smoking negated in part by increasing obesity and diabetes.

However, an ageing population will increase the CHD burden. Relative inequalities have persisted as CHD mortality rates have fallen more slowly in the most deprived areas [2]. Risk factor levels in England remain above both previous government targets and best levels achieved internationally. Inequalities in risk factors in England have likewise shown little or no improvement since 1994. Absolute inequalities in obesity and diabetes have widened, reflecting recent larger increases in the most deprived areas [3].

Estimating potential reductions in CHD deaths through decreases in modifiable risk factors would help policy-makers. In this modelling study, we assessed the potential contribution of risk factor reduction on future CHD mortality levels, and on absolute and relative inequalities, using the IMPACT_SEC_ model which quantifies the relative contributions of risk factor trends and treatment uptake to recent declines in CHD mortality in England [1]. Our base year was 2007 and the model was extended to 2020 to estimate the impact of four feasible risk factor change scenarios. 2020 was chosen as being sufficiently close to allow reasonable predictions and therefore be of direct policy relevance.

## Methods

### IMPACT_SEC_ model

The IMPACT_SEC_ model is described in detail elsewhere [1]. Briefly, the model quantifies the contribution of risk factor trends (smoking, systolic blood pressure (SBP), cholesterol, body mass index (BMI), diabetes, and physical activity) and changes in uptake of treatments to changes in CHD mortality rates in England. For this study we focussed exclusively on changes in risk factor levels in the population as a whole.

We used two validated methods to quantify the relationship between risk factor change and the consequent change in CHD mortality. First, the **regression method** is based on beta coefficients from large meta-analyses which summarise the independent mortality effects of changes in risk factors measured on a continuous scale - total cholesterol, BMI, and SBP [4–7]. We estimated the numbers of CHD deaths prevented/postponed (DPPs) in 2020 as a product of deaths expected in 2020 (assuming no change in CHD rates from 2007), absolute change in average risk factor levels between 2007 and 2020, and the beta coefficient. Second, **population attributable risk fractions** (PARF) calculated using Levin’s formula quantified the impact of change in three binary risk factors - current smoking, physical inactivity, and diabetes [8]. We calculated DPPs in 2020 as a product of deaths expected in 2020 and absolute change in PARF between 2007 and 2020.

### Estimating combined effects of risk factor change

Beta coefficients and relative risks were age- and gender-specific but were assumed equal across socioeconomic groups. The number of DPPs attributable to individual risk factors cannot be added due to overlap between them because of multi-causality and because the effects of some risk factors are partly mediated through other risk factors (e.g., the effects of BMI on CHD mortality are partly mediated through SBP and usual fasting plasma glucose) [9]. The combined impact of all risk factors was therefore estimated using the standard formula that accounts for multi-causality [1,10,11]. For example, given PARF values of 0.231, 0.286 and 0.021 for three risk factors the PARF formula accounting for multi-causality equals 1-((1-0.231)×(1-0.286)×(1-0.021)) = 0.462, a mortality reduction lower (i.e., more conservative) than the additive value of 0.538. The combined effect of change in all risk factors was calculated by age, gender, and socioeconomic group. Detailed information on our modelling approach is provided as supporting information in Text S1.

### Contribution of each risk factor to mortality gain

The percentage contribution of each risk factor to mortality gain was calculated separately for continuous and binary variables. The former equalled the relative change in CHD mortality associated with absolute change; the latter equalled absolute change in the PARF.

### Area deprivation, baseline mortality rates and projected population numbers

Area deprivation was defined using the Index of Multiple Deprivation (IMD) 2007 with small areas grouped into quintiles, ranked from Q1 (least deprived) to Q5 (most deprived) [12]. CHD (*International Classification of Diseases ICD-10: I20-I25*) deaths in 2007 by gender, age-group (in 10-y bands, 25-34 to age ≥ 85 years) and IMD quintile were obtained from the Office for National Statistics. Corresponding population denominators in 2020 were estimated using population projection methods.

### Estimating changes in absolute and relative inequalities by 2020

Expected deaths from CHD in 2020 were calculated assuming no change in gender-, age- and IMD quintile-specific mortality rates. Mortality rates in 2020 in each scenario were calculated by dividing expected CHD deaths *not* prevented by the 2020 population. We calculated age-standardised rate differences and rate ratios between Q1 and Q5 and their percentage reduction from 2007. Direct age-standardisation was computed using the European Standard Population.

### Risk factor data and risk factor change scenarios

Risk factor data from 1994 to 2008 were obtained from the Health Survey for England (HSE). Sampling methods and data collection instruments are described in detail elsewhere [1,3,13]. (See Table C in Text S1 for risk factor definitions.) 

Previous modelling studies quantified mortality gains from maximum risk factor reduction or achievement of “low-risk” levels [14,15]. Such aspirational scenarios appear unlikely and may therefore be less relevant to policy-makers. Instead, we defined four more realistic scenarios, based on recent national and international trends, and compared these to a baseline scenario of no change (Table 1). Each scenario is described below.

**Table 1:** Risk factor levels in England in 2007 and 2020 by risk factor change scenario.

**Risk factor**	**2007**	**2020 scenarios**			
	**Baseline**	**Worst case**	**Current trends**	**Intermediate**	**Optimal**
**Smoking (%)**					
All	22.9	22.9	16.8	16.5	10.0
Men	24.4	24.4	20.7	17.2	10.0
Women	21.5	21.5	12.9	15.7	10.0
**Physical inactivity (%)**					
All	74.6	74.6	65.8	52.3	30.0
Men	73.4	73.4	63.7	51.7	30.0
Women	75.8	75.8	67.9	52.9	30.0
**Diabetes (%)**					
All	5.4	15.0	11.3	4.7	4.0
Men	6.3	17.6	11.7	5.5	4.7
Women	4.4	12.4	10.8	3.9	3.3
**Systolic Blood Pressure (mmHg)**					
All	127.4	127.4	120.3	122.4	117.4
Men	130.8	130.8	126.6	125.8	120.8
Women	124.0	124.0	114.0	119.0	114.0
**Total cholesterol (mmol/l)**					
All	5.5	5.5	5.3	5.2	4.9
Men	5.5	5.5	5.3	5.2	4.9
Women	5.5	5.5	5.2	5.2	4.9
**Body mass index (kg/m^2^)**					
All	27.5	29.4	28.3	26.3	25.0
Men	27.8	29.6	28.6	26.8	25.9
Women	27.3	29.1	27.9	25.7	24.1

#### Baseline scenario. Assuming no change in risk factor levels or CHD mortality rates

Risk factor levels and CHD rates in 2007 would persist unchanged to 2020.

#### Scenario 1. Assuming current trends continue

Fractional polynomial functions to accommodate possible non-linearity were fitted to the HSE data with the risk factor as dependent variable and survey year as explanatory variable [16]. Best-fitting functions fitted to 1994-2008 data were projected forward to 2020. Separate models were fitted by gender, age and deprivation quintile. Due to small numbers we used an upper age band of ≥75 years.

#### Scenario 2. Achievement of optimal levels

Where possible we applied optimal levels observed internationally. SBP falls of ≥10 mmHg have been observed since 1980 in Finland, France, Iceland and Switzerland, and so a 10 mmHg fall was considered plausible for an optimal scenario [17]. Likewise, falls in total cholesterol of 0.6 mmol/l since 1980 have occurred in the US and several Nordic countries [18,19]. BMI levels were set at the lowest levels currently observed in Western Europe (men: 25.9 kg/m^2^ in France; women: 24.1 kg/m^2^ in Switzerland) [20]. For other risk factors, we applied target levels. Thus: reducing smoking levels to a target of 10% by 2020 was outlined in the previous government’s tobacco control strategy, *A Smokefree Future* [21]. *Game Plan*, the previous government’s strategy for delivering physical activity objectives, stated that 70% of adults should undertake ≥ 30 minutes of moderate-to-vigorous activities on at least five days a week by 2020 [22].

Finally, UK governments have not set targets to reduce diabetes levels which currently lie close to the European average of 5% [23]. Our optimal target was set at 4%, consistent with current levels in Sweden [23].

#### Scenario 3. Intermediate levels halfway between current and optimal levels

Achieving optimal levels represents a major challenge. Recent increases in BMI and diabetes, for example, must be successfully halted before being reduced to optimal levels. Hence we defined an intermediate scenario in which risk factor levels reach a point *halfway* between current and optimal levels, as defined above.

#### Scenario 4. Worst case scenario: plateauing or worsening trends

Levelling-off in SBP declines was recently observed in the US and Finland and so we assumed no change in mean SBP levels [20,24]. Cholesterol levels in younger age groups in the US have stabilised, therefore no change was also assumed for total cholesterol [20]. Recent plateauing in obesity [25] is not inevitable and so we assumed annual increases in BMI of 0.5%, similar to increases in the US over 1990-2005 [26]. Increasing diabetes levels in England in older groups reflect BMI increases and improved case-ascertainment. The APHO Diabetes Prevalence Model 2020 estimate is 8.5% reflecting population change and BMI increases [27] - but such models may underestimate likely improvements in survival for diabetic people [28]. Diabetes levels were therefore assumed to reach 15% in 2020 (from 5% at baseline).

### Deprivation gradients in risk factor levels

Previous studies examined mortality gains through: (1) equal absolute change in risk factors across social groups, (2) improvement in least advantaged groups to levels achieved in the most advantaged, and (3) proportional change (optimal levels achieved overall but with no change in socioeconomic gradients) [14,29,30]. We assumed proportional change in risk factor levels in optimal, intermediate, and worst case scenarios and used IMD quintile specific projections in the current trends scenario.

### Uncertainty analysis

Monte Carlo simulation using the Excel add-in Ersatz was used to compute 95% uncertainty intervals (UIs) of deaths prevented in each scenario. This involved replacing all fixed input parameters by appropriate probability distributions, and repeatedly recalculating model outputs with values sampled from the defined input distributions [31]. 95% UIs using the 2.5^th^ and 97.5^th^ percentiles were generated from 1000 model iterations.

### Ethics statement

Each sampled address for the Health Survey for England is sent an advance letter which introduces the survey and states that an interviewer would be calling to seek permission to interview. A leaflet is also enclosed providing general information about the survey and some of the findings from previous surveys. Individual interviews are conducted with adults who give verbal informed consent. At the end of individual interviews, participants are asked for agreement to a follow-up visit by a trained nurse. Written consent is obtained for collection of non-fasting blood samples.

There is no formal record that participants have given verbal consent to the individual interview or give physical measurements that are not biological samples (e.g., height, weight, and blood pressure). It is made clear in the advance letters and information leaflets that participation in the survey is entirely voluntary, and that participants may decline to answer individual questions, withdraw or stop at any time, or refuse any particular measurement if they wish to do so. Interviewers and nurses will often repeat this information in their introductions and when they are setting up appointments, and throughout the interview as necessary. Indeed, many individuals do refuse to participate in the survey: others may refuse individual questions, decline to continue part way through an interview or refuse physical measurements. It is also standard practice to conduct interviews and nurse visits some time after an appointment has been made so that individuals have to a chance to reflect on their agreement before the appointment takes place.

The procedures used in the Health Survey for England to obtain informed consent are very closely scrutinised by a National Health Service (NHS) ethics committee *each year*. Information leaflets and both the content and wording of questionnaires are also carefully reviewed by the ethics committees.

All Local Research Ethics Committees (LREC) in England were approached for ethical approval of the 1994 survey before it started. All gave their approval, with the exception of Shropshire and Northallerton. Sampled addresses within these areas were withdrawn from the survey. In addition, agreement could not be reached with East Birmingham LREC in time to cover selected addresses during the first quarter of fieldwork. These addresses therefore were not covered.

All LRECs in England were approached for ethical approval for the 1995 survey before it started. All gave their approval, with the exception of Shropshire and the Isle of Wight. Sampled addresses within these areas were withdrawn from the survey. All LRECs in England, with two exceptions, were approached for ethical approval in both 1996 and 1997. All gave their approval. As the 1996 and 1997 designs were similar to that of 1995, agreement from the Shropshire and Isle of Wight LRECs was not sought for either survey. The areas covered by these two committees were omitted from the sample selection process.

Ethical approval for each survey between 1998 and 2001 was obtained from the North Thames Multi-centre Research Ethics Committee (MREC) and from all LRECs in England; approval for each survey between 2002 and 2007 was obtained from the London Multi-Centre Research Ethics Committee. Ethical approval for the 2008 survey was obtained from the Oxford A Research Ethics Committee (reference number 07/H0604/102).

Our study, pooling Health Survey for England data from 1994–2008 to inform projections for the current trends scenario, is a secondary analysis of previously collected data and so additional ethical approval was not required.

## Results

**Figure 1:**
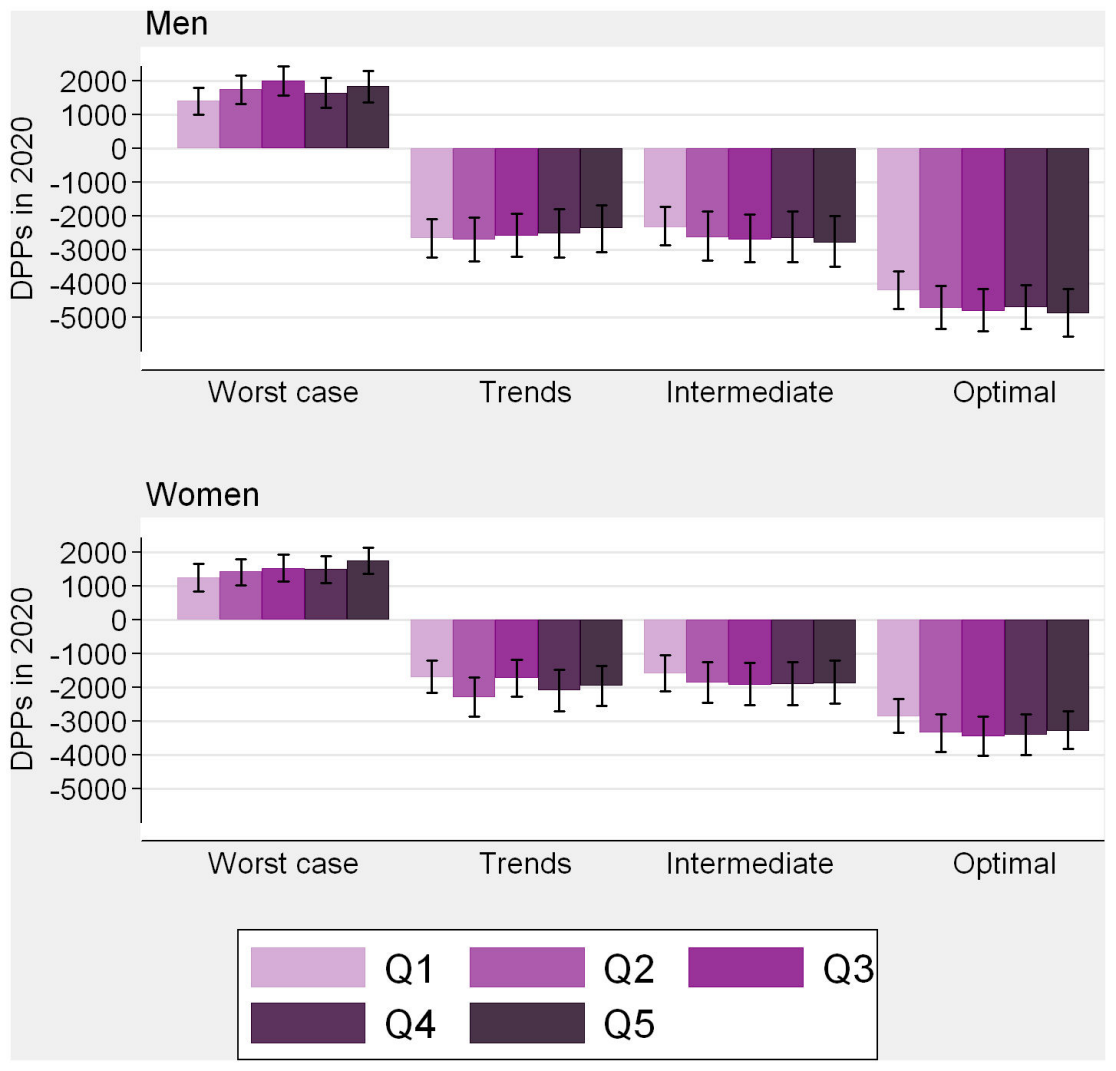
Reduction in CHD deaths in 2020 by risk factor change scenario. DPPs, deaths prevented or postponed. Q1 = least deprived; Q5 = most deprived. Positive DPPs show additional deaths representing adverse risk factor trends; negative DPPs show risk factor reduction. 95% uncertainty intervals shown by I bars.

Table 1 shows risk factor levels in 2007 and 2020 for each scenario. Socioeconomic gradients in 2007 were strongest for smoking, diabetes, physical activity, and BMI in women (Table E in Text S1). Table 2 shows the CHD deaths prevented/postponed (DPPs) in each scenario and Figure 1 shows accompanying 95% uncertainty intervals; Table 3 shows the estimated changes in CHD mortality rates over the 13-y period (For age-specific results see Tables K–N in Text S1).

**Table 2:** CHD deaths prevented/postponed in 2020 by risk factor change scenario.

	**Population (per 1000)**	**CHD mortality rate (per 100,000)** ^a^	**Expected deaths**	**Deaths prevented/postponed (DPPs)^b^**			
	**2020**	**2007**	**2020**	**2020**			
				**Worst case**	**Current trends**	**Intermediate**	**Optimal**
**All**							
**England** ^c^	39,787	147.4	97,059	16,170	-22,640	-22,330	-39,720
Q1	8,651	108.9	18,284	2,670	-4,370	-3,930	-7,070
Q2	8,476	125.1	20,382	3,190	-5,000	-4,500	-8,060
Q3	8,182	141.8	20,579	3,540	-4,330	-4,650	-8,280
Q4	7,633	169.1	19,483	3,160	-4,610	-4,580	-8,120
Q5	6,846	214.0	18,331	3,610	-4,320	-4,690	-8,200
**Men**							
**England** ^c^	19,462	200.3	57,270	8,700	-12,820	-13,140	-23,340
Q1	4,209	148.1	10,998	1,410	-2,660	-2,340	-4,210
Q2	4,130	169.5	12,021	1,770	-2,700	-2,640	-4,720
Q3	4,001	192.6	12,021	2,010	-2,590	-2,710	-4,820
Q4	3,753	230.2	11,283	1,650	-2,510	-2,660	-4,700
Q5	3,369	292.6	10,946	1,860	-2,350	-2,800	-4,890
**Women**							
**England** ^c^	20,325	94.6	39,789	7,470	-9,820	-9,190	-16,380
Q1	4,442	69.7	7,287	1,260	-1,710	-1,590	-2,860
Q2	4,345	80.8	8,361	1,420	-2,300	-1,860	-3,340
Q3	4,180	91.0	8,557	1,530	-1,740	-1,940	-3,460
Q4	3,881	108.0	8,200	1,510	-2,100	-1,920	-3,420
Q5	3,477	135.5	7,384	1,750	-1,970	-1,890	-3,310

^a^ Age-standardised.

^b^ DPPs rounded to nearest 10: positive DPPs represent adverse trends; negative DPPs show risk factor reduction.

^c^ Summation of IMD quintile counts.

**Table 3:** 2020 mortality rates by risk factor change scenario.

**Scenarios**	**CHD mortality rate in 2020 (per 100,000)** ^a^						**Relative change (%)** ^b^					
	**England** ^c^	**Q1**	**Q2**	**Q3**	**Q4**	**Q5**	**England** ^c^	**Q1**	**Q2**	**Q3**	**Q4**	**Q5**
**All**												
Baseline_2007_	147.4	108.9	125.1	141.8	169.1	214.0	−	−	−	−	−	−
Worst case	171.5	124.7	144.4	165.8	197.0	256.2	16.9	15.2	15.8	17.2	17.2	20.7
Current trends	113.0	83.3	93.9	110.2	128.9	162.2	-23.8	-23.6	-26.2	-22.7	-23.9	-24.5
Intermediate	110.5	83.3	94.7	106.2	124.7	153.6	-25.1	-23.5	-24.4	-25.1	-26.4	-28.4
Optimal	82.9	63.6	71.5	79.5	91.7	110.0	-43.9	-41.7	-43.0	-44.1	-45.9	-48.8
**Men**												
Baseline_2007_	200.3	148.1	169.5	192.6	230.2	292.6	−	−	−	−	−	−
Worst case	230.9	167.6	194.4	224.4	265.2	344.9	15.2	13.2	14.7	16.5	15.2	17.9
Current trends	155.1	113.5	131.0	151.3	175.9	223.1	-22.6	-23.3	-22.7	-21.4	-23.6	-23.7
Intermediate	150.4	113.5	128.5	144.6	170.1	210.8	-24.9	-23.3	-24.2	-24.9	-26.1	-28.0
Optimal	113.1	86.9	97.3	108.5	125.5	151.5	-43.5	-41.3	-42.6	-43.6	-45.5	-48.2
**Women**												
Baseline_2007_	94.6	69.7	80.8	91.0	108.0	135.5	−	−	−	−	−	−
Worst case	112.2	81.7	94.4	107.3	128.8	167.4	18.6	17.3	16.9	17.9	19.3	23.6
Current trends	71.0	53.1	56.8	69.1	82.0	101.2	-24.9	-23.8	-29.7	-24.0	-24.1	-25.3
Intermediate	70.6	53.2	60.9	67.9	79.2	96.4	-25.4	-23.7	-24.6	-25.4	-26.7	-28.8
Optimal	52.6	40.3	45.7	50.5	57.9	68.4	-44.4	-42.1	-43.4	-44.5	-46.4	-49.5

^a^ Age-standardised.

^b^ Positive values represent increasing mortality rates; negative values show declines.

^c^ Expected deaths and DPPs summed over IMD quintiles.

### Estimated CHD mortality in 2020 based on different scenarios

#### Baseline scenario. Assuming no change in risk factor levels or CHD mortality rates

Approximately 97,000 CHD deaths would be expected in 2020. This would represent 30% more deaths than in 2007, simply reflecting population ageing (Table 2).

#### Scenario 1. Assuming current trends continue

Approximately 22,640 (95% *uncertainty interval*: *20,390-24,980*) fewer CHD deaths would occur in 2020 should current risk factor trends continue compared to the baseline of no change: 12,820 in men (*11,140-14,480*) and 9,820 in women (*8,230-11,360*). This would represent the net of 28,800 deaths prevented due to falls in smoking, physical inactivity, SBP and total cholesterol negated by 6,160 additional deaths through increases in BMI and diabetes. Relative reductions in CHD mortality rates would be broadly similar across IMD quintiles (men: 23% in Q1 and 24% in Q5; women: 24% in Q1 and 25% in Q5) (Table 3).

#### Scenario 2. Achievement of optimal levels

Achieving optimal levels would prevent approximately 39,720 deaths (*37,120-41,900*). 23,340 deaths would be averted in men (*21,390-25,100*); 16,380 in women (*14,740-17,820*). DPPs would be highest in the most deprived areas (men: 4,210 in Q1 and 4,890 in Q5; women: 2,860 in Q1 and 3,310 in Q5). CHD mortality rates would fall to 45% of 2007 levels, with relative reductions just below 50% in both sexes in Q5 compared with 41% in men and 42% in women in Q1 (Table 3).

#### Scenario 3. Intermediate levels halfway between current and optimal levels

Deaths prevented in the intermediate scenario would be similar to the current trends scenario (22,330 (19,850-24,300) fewer deaths). The pattern of risk factor change would however be very different. The intermediate scenario assumed: (1) falls, rather than increases, in BMI and diabetes; (2) larger reductions in smoking and physical inactivity; and (3) more modest falls in SBP and cholesterol.

#### Scenario 4.Worst case scenario: plateauing or worsening trends

Roughly 16,170 (13,880-18,420) additional CHD deaths would be expected in the worst case scenario: 8,700 in men (*7,190-10,030*) and 7,470 in women (*5,800-9,270*), reflecting continuing increases in BMI and diabetes. Assuming proportional change in BMI and diabetes across IMD quintiles implies larger increases in absolute terms in quintiles with the highest baseline levels. As BMI and diabetes are socially patterned, especially in women, additional deaths would be larger in the most deprived areas: 3,610 in Q5 (*3,040-4,200*) compared with 2,670 in Q1 (*2,120-3,210*).

### Changes in absolute and relative inequalities by 2020

Table **4** shows the baseline CHD rates in Q1 and Q5 and the percentage reduction in age-standardised rate differences and rate ratios. Assuming current risk factor trends continue the absolute gap in rates between Q1 and Q5 would fall by approximately 25% (men: 24%; women: 27%). However the reduction in rate ratios would be negligible (1% and 4% in men and women, respectively). Achieving optimal levels would reduce absolute differences by 55% in men and 57% in women. Rate ratios would fall by 24% and 26% in men and women, respectively. Rate differences in the intermediate scenario would fall by roughly 33%, with a 13% reduction in the rate ratio. Absolute disparities in CHD rates under the worst case scenario would increase by 23% and 30%, whilst inequalities in relative terms would increase by 9% and 11% in men and women, respectively.

**Table 4:** Changes in inequalities by risk factor change scenario.

**Scenarios**	**CHD mortality rate** ^a^			**Inequalities**			
	**In 2020 (per 100,000)**			**Absolute**		**Relative**	
	**England** ^b^	**Q1**	**Q5**	**Difference**	**Change (%)** ^c^	**Ratio**	**Change (%)** ^c^
**Men**							
Baseline_2007_	200.3	148.1	292.6	144.5	−	1.98	−
Worst case	230.9	167.6	344.9	177.3	-22.7	2.06	-8.5
Current trends	155.1	113.5	223.1	109.6	24.1	1.97	1.0
Intermediate	150.4	113.5	210.8	97.2	32.7	1.86	12.2
Optimal	113.1	86.9	151.5	64.6	55.3	1.74	23.8
**Women**							
Baseline_2007_	94.6	69.7	135.5	65.8	−	1.94	−
Worst-case	112.2	81.7	167.4	85.7	-30.3	2.05	-11.1
Current trends	71.0	53.1	101.2	48.1	26.9	1.91	4.1
Intermediate	70.6	53.2	96.4	43.3	34.2	1.81	13.8
Optimal	52.6	40.3	68.4	28.1	57.3	1.70	26.2

^a^ Age-standardised rates.

^b^ Calculated using expected deaths and DPPs summed over IMD quintiles.

^c^ Positive values show inequality reductions; negative values show increases.

### Contribution of each risk factor to mortality gain

**Figure 2:**
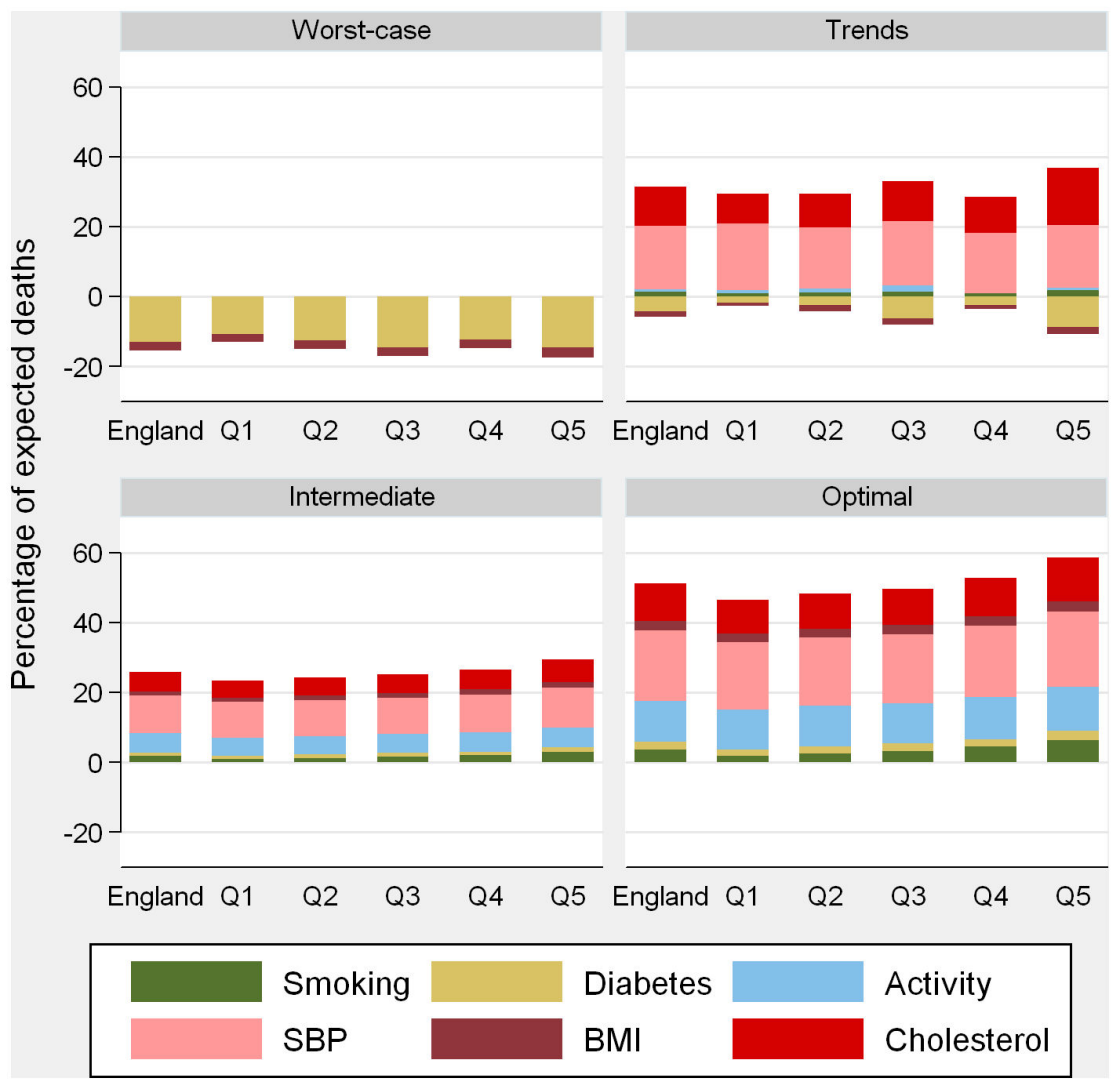
Risk factor contributions to mortality gains in 2020 in men by risk factor change scenario. Q1 = least deprived; Q5 = most deprived. SBP: systolic blood pressure; BMI: body mass index.

Figure 2 (men) and 3 (women) show the contribution of each risk factor to CHD mortality change. SBP falls would provide the largest contribution, with benefits spread equitably across IMD quintiles. Due to the current social patterning, larger absolute declines in smoking, diabetes and BMI (especially in women) in the optimal and intermediate scenarios would achieve larger benefits in the most deprived areas. In the optimal scenario, falls in SBP would reduce CHD deaths by roughly 20% in both sexes; achieving the physical activity target would reduce CHD deaths by 12% (in both sexes) and 10% would be averted by declines in total cholesterol (11% and 8% in men and women, respectively).

**Figure 3:**
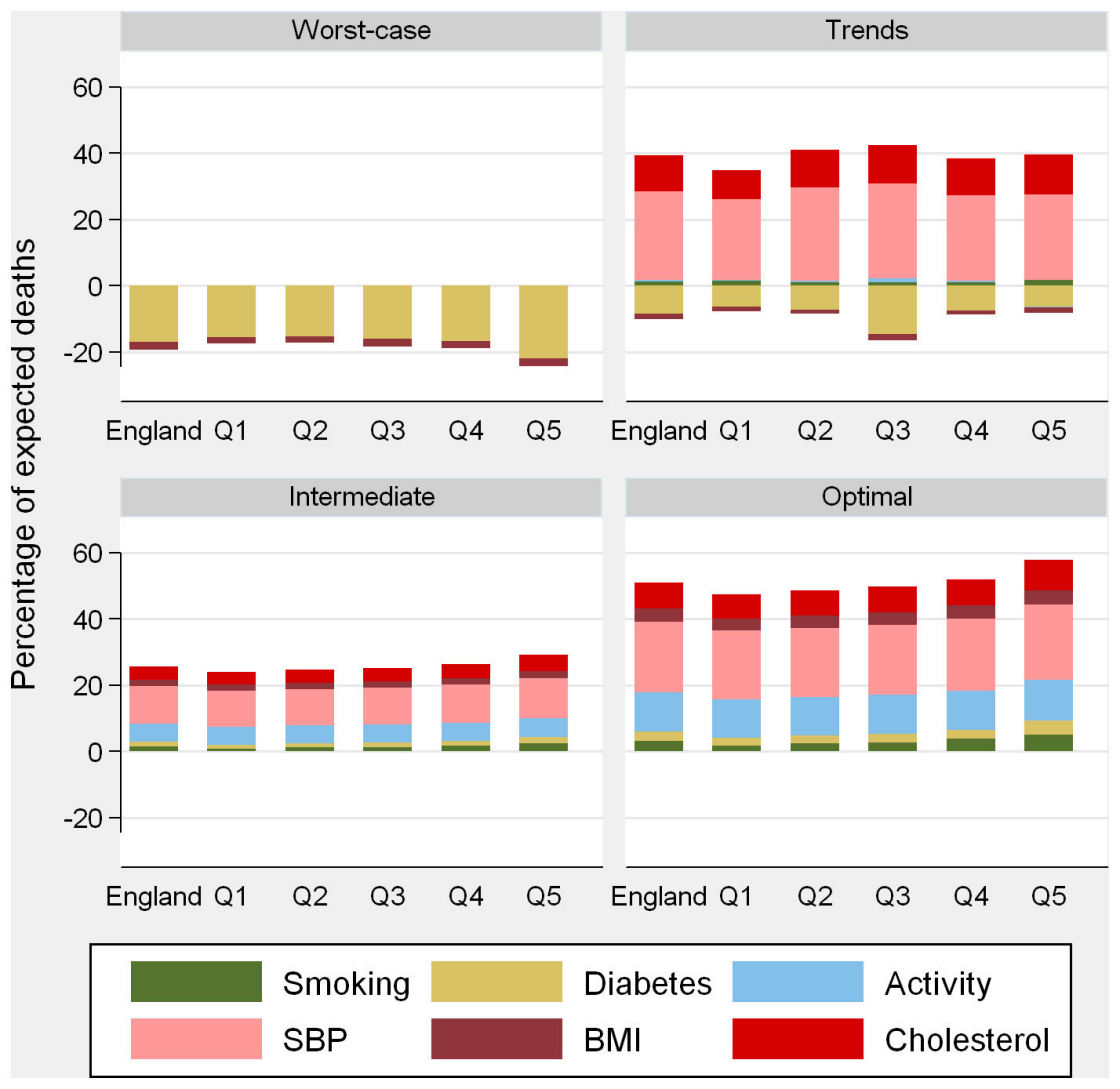
Risk factor contributions to mortality gains in 2020 in women by risk factor change scenario. Q1 = least deprived; Q5 = most deprived. SBP: systolic blood pressure; BMI: body mass index.

## Discussion

We estimated the impact of four realistic risk factor scenarios on levels of CHD mortality and inequalities in England in 2020. Population ageing would result in approximately 23,000 additional CHD deaths if risk factors remain at 2007 levels. But, if recent risk factor trends continue, nearly 23,000 deaths might be averted. In the optimal scenario, approximately 40,000 deaths might be prevented, reducing inequalities by 56% in absolute terms and 25% in relative terms. The intermediate scenario would prevent a similar number of deaths as the current trends scenario but would reduce inequalities more with reductions of 33% and 13% in absolute and relative terms, respectively. Over 16,000 additional deaths with increases in rate disparities would be anticipated in the worst case scenario.

### Comparisons with other studies

Our conclusions correspond with previous modelling studies. A recent US study considered proportional achievement of Healthy People 2010 targets [30]. Absolute falls in the 10-year risk of CHD mortality would be 28% in both sexes, with relative reductions 9%. Inequalities in absolute terms would fall by 35% in men and 47% in women; relative inequalities would fall by 17% and 30%. A UK prospective cohort study (Whitehall study of male civil servants) considered reductions in the 15-year risk of CHD mortality under two scenarios: (1) implementing best-practice interventions in high and low employment grades, and (2) primordial prevention (low lifetime risk factor levels) [14]. CHD mortality in the two scenarios would fall by 57% and 73%. Absolute differences in risk would decrease by 69% and 86%; reductions in relative terms by 30% and 53%.

The larger reductions in absolute rather than relative inequalities in our study arise from two main reasons. First, given the persistent inequalities in risk factor levels in England since the early 1990s we assumed proportional change in our scenarios, i.e. equalising relative change across IMD quintiles while varying absolute change. Second, shifting the entire population distribution in risk factors that are not socially patterned can still powerfully reduce inequalities as absolute risk reduction will be greatest in groups with the highest baseline mortality rates [32]. This is especially relevant in England as our previous analyses found no social patterning in mean levels of SBP and total cholesterol - two of the three most powerful risk factors for cardiovascular disease [3].

Choosing different scenarios would have produced different results. Levelling-up risk factor levels in the most deprived areas to current levels in the least deprived implies equal reductions in absolute and relative inequalities [30] – but such a scenario seems unrealistic. Targeting improvement in the most deprived areas would impact strongly on absolute and relative inequalities but would have modest impact on overall mortality levels [30]. Furthermore, this scenario, implying an uneven distribution of resources, might not be accepted by those living in less deprived areas.

### Strengths and limitations

The IMPACT_SEC_ model has successfully explained recent change in CHD mortality across age, gender and deprivation quintiles in England, and in other countries and over different time periods. Previous work by the authors showed that the model parameters had face validity in that the model explained approximately 90% of the observed decline in CHD mortality between 2000 and 2007 [1]. Our aim in this study was not to provide a definitive prediction of future CHD mortality levels but rather to use an established model to explore the mortality impact of different but plausible scenarios of risk factor reduction.

We used UK and international risk factor trend data to inform our scenarios and we summarised potential inequality reductions in both absolute and relative terms. The findings however need to be considered in light of our key modelling assumptions.

Beta coefficients and relative risks (RRs) were assumed to accurately reflect causality [29]. The estimates of RRs used as input to the PARF calculations were developed by expert working groups for the World Health Organization’s Global Burden of Disease 2001 study and were adjusted for correlation between risk factors, confounding and mediation [33,34]. The standard formula used to account for multi-causality assumes that: (1) exposures to risks are uncorrelated, and that (2) the effect of one risk factor is not mediated through others [15]. The latter assumption was partly corrected for through using beta coefficients/RRs adjusted for confounding effects. However, residual effects may remain [35]. Double-counting of mortality gains through multi-causality is therefore likely to have remained in our model to some degree.

It is important to distinguish between a greater CHD burden in the most deprived areas reflecting higher levels of risk factors for cardiovascular disease (smoking, physical inactivity, diabetes and BMI), and effect-modification, whereby the impact of risk factors may systematically vary across areas with different deprivation levels. Consistent with recent Health Survey for England data, our modelling approach allowed risk factor levels in the baseline year to vary across deprivation quintiles. Our assumption of proportional risk factor change meant that the magnitude of falls in risk factor levels over the 13-yr period, in absolute terms, was greater in areas with the highest baseline levels. However, as in other recent modelling studies, we have assumed that the percentage decrease in CHD mortality per unit change in risk factor levels was equal across deprivation quintiles. This assumption was made due to the lack of systematic data on whether, and how, the impact of proximate risk factors on mortality differs by socioeconomic group. We acknowledge that bias in our model through assuming equal beta coefficients/RRs across deprivation quintiles remains possible, but that such bias is unlikely to be very large. For example, two recent collaborative analyses found that the impact of BMI and diabetes on cardiovascular disease and all-cause mortality respectively showed no appreciable reduction in age- and gender-adjusted hazard ratios after additional adjustment for indicators of socioeconomic status [36,37].

Finally, a geographical index of socioeconomic position (SEP) was used, the Index of Multiple Deprivation (IMD) 2007. Area-level categorizations of socioeconomic status are not perfect proxies for individual-level SEP. However, assigning individuals to individual-level measures of SEP is not without its problems. In England, individual-level SEP categories have changed over time and are particularly problematic for older people (where the majority of CHD deaths occur). For example, individual-level measures of social position for those aged 65 and over is not reliably recorded in death certificates [38], whereas residential address recording is complete and accurate. Area-level measures of SEP also help to capture the contextual effects of living conditions [39].

It is likely that some areas will undergo ‘gentrification’ while others move down the deprivation ladder. In previous work we tested the assumption of stability in the *relative* ranking of deprivation quintile allocation in England across a 20-yr period (1981-2001) [2,40]. Our results showed that the top and bottom fifths of the deprivation distribution remained stable over the two decades. Just over three-quarters (76%) of the population in 1981 who were living in either the least or most deprived quintiles remained in their respective top/bottom quintiles in 2001. Hence we would anticipate changes in the deprivation levels of areas over the time period of our study, but no dramatic change in the relative stability of quintile membership.

## Conclusions

This population-based modelling suggests that at best, deaths from CHD in 2020 could be reduced by approximately 45% of the level in 2007, with the largest absolute gains occurring in the most deprived areas.

## Supporting Information

Text S1 **Appendix and supporting tables**. In the appendix we provide more details on the modelling techniques used and provide supporting tables showing age-specific results. Contents are as follows. Table A: Beta coefficients for major risk factors. Table B: Relative risks for CHD for smoking, diabetes and physical inactivity. Table C: Risk factor definitions from the Health Survey for England and participants at each stage. Table D: Risk factor levels in the Health Survey for England using pooled data (2003-08) by age-group, gender and deprivation quintile. Table E: Smoothed baseline (2007) risk factor levels by age-group, gender and deprivation quintile. Table F: Worst-case scenario: risk factor levels by age-group, gender and deprivation quintile. Table G: Assuming current trends continue: risk factor levels by age-group, gender and deprivation quintile. Table H: Intermediate scenario (halfway between current and optimal): risk factor levels by age-group, gender and deprivation quintile. Table I: Optimal scenario: risk factor levels by age-group, gender and deprivation quintile. Table J: Population in 2020, baseline mortality rates, and expected deaths assuming no change in CHD mortality rates by age-group, gender and deprivation quintile. Table K: Deaths prevented/postponed in each scenario by age-group, gender and deprivation quintile. Table L: Deaths prevented/postponed with 95% uncertainty intervals in each scenario by gender and deprivation quintile. Table M: Expected CHD mortality rates per 100,000 in each scenario by age-group, gender and deprivation quintile. Table N: Relative change in CHD mortality (%) in each scenario by age-group, gender and deprivation quintile.
(DOCX)
