# Supplementary material for: Quantifying Policy Options for Reducing Future Coronary Heart Disease Mortality in England: A Modelling Study
Source: PLoS One. 2013 Jul 25;8(7):e69935. doi: 10.1371/journal.pone.0069935 (PMC3723729; doi:10.1371/journal.pone.0069935)
Supplement: Text S1 — Appendix and supporting tables. In the appendix we provide more details on the modelling techniques used and provide supporting tables showing age-specific results. Contents are as follows. Table A: Beta coefficients for major risk factors. Table B: Relative risks for CHD for smoking, diabetes and physical inactivity. Table C: Risk factor definitions from the Health Survey for England and participants at each stage. Table D: Risk factor levels in the Health Survey for England using pooled data (2003-08) by age-group, gender and deprivation quintile. Table E: Smoothed baseline (2007) risk factor levels by age-group, gender and deprivation quintile. Table F: Worst-case scenario: risk factor levels by age-group, gender and deprivation quintile. Table G: Assuming current trends continue: risk factor levels by age-group, gender and deprivation quintile. Table H: Intermediate scenario (halfway between current and optimal): risk factor levels by age-group, gender and deprivation quintile. Table I: Optimal scenario: risk factor levels by age-group, gender and deprivation quintile. Table J: Population in 2020, baseline mortality rates, and expected deaths assuming no change in CHD mortality rates by age-group, gender and deprivation quintile. Table K: Deaths prevented/postponed in each scenario by age-group, gender and deprivation quintile. Table L: Deaths prevented/postponed with 95% uncertainty intervals in each scenario by gender and deprivation quintile. Table M: Expected CHD mortality rates per 100,000 in each scenario by age-group, gender and deprivation quintile. Table N: Relative change in CHD mortality (%) in each scenario by age-group, gender and deprivation quintile. (DOCX) [file pone.0069935.s001.docx]

# Supporting information:

# Quantifying Policy Options for Reducing Future Coronary Heart Disease Mortality in England: A Modelling Study

Shaun Scholes, Madhavi Bajekal, Paul Norman, Martin O’Flaherty, Nathaniel Hawkins, Mika Kivimäki, Simon Capewell, Rosalind Raine

# *Population counts in 2020 by age, gender and area deprivation quintiles*

A time-series of age-sex populations for Lower Super Output Areas (LSOAs) were estimated for the 1981 to 2000 period with 2001-2007 estimates obtained from the Office for National Statistics (ONS). All LSOA estimates were constrained to be consistent with ONS produced local government district estimates and then aggregated into Index of Multiple Deprivation 2007 quintiles. Curves were fitted to each age-sex count by deprivation quintile to project the direction of population change to 2020. This approach picks up cohort effects in the population with respect to growth\contraction and the directions of change in relation to deprivation since life-course moves between area-types are typical. Projected counts were constrained to ONS population projections for England.

***Deaths from CHD prevented or postponed through risk factor change***

Two validated methods were used to describe the relationships between population changes in risk factors and the consequent change in population CHD mortality rates between 2007 (baseline year) and 2020 (our chosen final year).

The first is a **regression method:** beta coefficients from large meta-analyses and cohort studies were used to assess the mortality effects of changes in total cholesterol, body mass index (BMI) and systolic blood pressure (SBP).^1-4^ Each beta coefficient quantifies the independent (log-linear) relationship between absolute change in risk factor levels between two points in time and the consequent relative change in CHD mortality rates. The subsequent number of CHD deaths prevented/postponed (DPPs) in 2020 compared with those expected in 2020 assuming no change in CHD rates from 2007 was estimated as follows:

*Number of CHD DPPs in 2020 = number of CHD deaths expected in 2020 (assuming rates in 2007 remain unchanged) × 1- exponential(beta coefficient × absolute change in mean risk factor levels_2020-2007_)*

The second method, **population attributable risk fractions** (PARF) was used to assess the impact of changes in three binary risk factors: current smoking, physical activity, and diabetes. PARF was calculated in the standard way using Levin’s formula^5^:

PARF = [P × (RR-1)] / [1 + P × (RR-1)]

where P denotes prevalence and RR the relative risk in CHD mortality associated with risk factor presence. DPPs in 2020 were estimated as the product of the CHD deaths expected in 2020 (assuming no change in mortality rates from 2007) multiplied by the absolute change in PARF between 2007 and 2020.

Beta coefficients for continuous risk factors taken from large meta-analyses and cohort studies are shown in **Table A**; relative risks for dichotomous risk factors in **Table B**. Further details are provided in the supporting information for the IMPACT_SEC_ model.^6^

**Table A. Beta coefficients for major risk factors**

*Estimated β coefficients from multiple regression analyses for the relationship between absolute changes in population mean risk factors and % change in coronary heart disease mortality rates for men and women, stratified by age. Data sources and values.*

| **Systolic blood pressure** | **Age group (years)** | | | | |
| --- | --- | --- | --- | --- | --- |
|  | **25-44** | **45-54** | **55-64** | **65-74** | **≥75** |
| **Men** (hazard ratio per 20 mmHg) | 0.49 | 0.49 | 0.52 | 0.58 | 0.65 |
| **Men** (log hazard ratio per 1 mmHg) | **-0.036** | **-0.035** | **-0.032** | **-0.027** | **-0.021** |
| *Minimum* | *-0.029* | *-0.028* | *-0.026* | *-0.022* | *-0.017* |
| *Maximum* | *-0.043* | *-0.042* | *-0.039* | *-0.032* | *-0.025* |
| **Women** (hazard ratio per 20 mmHg) | 0.40 | 0.40 | 0.49 | 0.52 | 0.59 |
| **Women** (log hazard ratio per 1 mmHg) | **-0.046** | **-0.046** | **-0.035** | **-0.032** | **-0.026** |
| *Minimum* | *-0.037* | *-0.037* | *-0.028* | *-0.026* | *-0.021* |
| *Maximum* | *-0.055* | *-0.055* | *-0.042* | *-0.039* | *-0.031* |
| Source: Prospective studies collaborative meta-analysis, Lancet 2002.^4^  Units: Percentage change in CHD mortality per 1/20 mmHg change | | | | | |

| **Total cholesterol** | **Age group (years)** | | | | | |
| --- | --- | --- | --- | --- | --- | --- |
|  | **25-44** | **45-54** | **55-64** | **65-74** | **75-84** | **≥85** |
| Mortality reduction per 1 mmol/l | | | | | | |
| **Men** | 0.55 | 0.53 | 0.36 | 0.21 | 0.21 | 0.21 |
| **Women** | 0.57 | 0.52 | 0.35 | 0.23 | 0.23 | 0.23 |
| Log coefficient |  |  |  |  |  |  |
| **Men** | **-0.799** | **-0.755** | **-0.446** | **-0.236** | **-0.117** | **-0.083** |
| *Minimum* | *-0.639* | *-0.604* | *-0.357* | *-0.189* | *-0.093* | *-0.067* |
| *Maximum* | *-0.958* | *-0.906* | *-0.536* | *-0.283* | *-0.140* | *-0.100* |
| **Women** | **-0.844** | **-0.734** | **-0.431** | **-0.261** | **-0.174** | **-0.051** |
| *Minimum* | *-0.675* | *-0.587* | *-0.345* | *-0.209* | *-0.139* | *-0.041* |
| *Maximum* | *-1.013* | *-0.881* | *-0.517* | *-0.314* | *-0.209* | *-0.062* |
| Source: Prospective studies collaborative meta-analysis, Lancet 2007.^1^  Units: Percentage change in CHD mortality per 1 mmol/l change in total cholesterol | | | | | | |

| **Body Mass Index (BMI)** | **Age group (years)** | | | | |
| --- | --- | --- | --- | --- | --- |
|  | **<44** | **45-59** | **60-69** | **70-79** | **≥80** |
| *James et.al (2004)*^3^ |  |  |  |  |  |
| Hazard ratio | 0.89 | 0.91 | 0.95 | 0.96 | 0.97 |
| Risk reduction^†^ per 1 kg/m^2^ | 0.11 | 0.09 | 0.05 | 0.04 | 0.03 |
| Age gradient (45-59 as reference) | 1.22 | -- | 0.56 | 0.44 | 0.33 |
| *Bogers (2006)*^2^ |  |  |  |  |  |
| Relative risks, CHD deaths per 5 kg/m^2^ |  | **1.16** |  |  |  |
| Relative risks per 1 kg/m^2^ (age gradients) | 1.04 | 1.03 | 1.02 | 1.01 | 1.01 |
| Log coefficients | **0.0363** | **0.0297** | **0.0165** | **0.0132** | **0.0099** |
| *Minimum* | *0.0255* | *0.0209* | *0.0116* | *0.0093* | *0.0070* |
| *Maximum* | *0.0466* | *0.0381* | *0.0212* | *0.0169* | *0.0127* |
| Source: Bogers et.al^2^, James et.al^3^  Units: Percentage change in CHD mortality per 1 kg/m^2^ change in BMI | | | | | |

† Risk reduction = 1 – hazard ratio

**Table B. Relative risks for CHD for smoking, diabetes and physical inactivity**

Relative risks (RRs) estimated by expert working groups for the World Health Organization’s (WHO) Global Burden of Disease (GBD) 2001 Study were used for smoking and physical activity.^7^ Effect estimates were based on systematic reviews of cohort studies (adjusted for regression dilution bias) and meta-analyses of randomised controlled trials. Age-variation in the relative risks for diabetes were taken from the DECODE study.^8^ These were then applied to the gender-variation in relative risks estimated by Huxley et.al.^9^ The set of RRs used for the three binary risk factors with 95% Confidence Intervals (in parentheses) are shown below. RRs were assumed in our modelling study to be constant across IMD quintiles.

|  | **Smoking** | **Physical inactivity** | **Diabetes** |
| --- | --- | --- | --- |
| **Men 25-34** | 5.51 (2.47-12.25) | 1.50 (1.35-1.67) | 4.33 (3.47-5.20) |
| **Men 35-44** | 5.51 (2.47-12.25) | 1.50 (1.35-1.67) | 3.22 (2.58-3.86) |
| **Men 45-54** | 3.04 (2.66-3.48) | 1.50 (1.35-1.67) | 2.14 (1.71-2.57) |
| **Men 55-64** | 2.51 (2.22-2.84) | 1.50 (1.35-1.67) | 1.99 (1.59-2.39) |
| **Men 65-74** | 1.69 (1.52-1.89) | 1.44 (1.30-1.61) | 1.86 (1.49-2.23) |
| **Men 75-84** | 1.31 (1.11-1.56) | 1.32 (1.19-1.47) | 1.71 (1.37-2.05) |
| **Men ≥85** | 1.05 (0.78-1.43) | 1.23 (1.11-1.37) | 1.71 (1.37-2.05) |
|  |  |  |  |
| **Women 25-34** | 2.26 (0.83-6.14) | 1.50 (1.35-1.68) | 7.55 (6.04-9.06) |
| **Women 35-44** | 2.26 (0.83-6.14) | 1.50 (1.35-1.68) | 5.63 (4.51-6.76) |
| **Women 45-54** | 3.78 (3.10-4.62) | 1.50 (1.35-1.68) | 3.81 (3.05-4.57) |
| **Women 55-64** | 3.21 (2.70-3.82) | 1.50 (1.35-1.68) | 3.12 (2.50-3.74) |
| **Women 65-74** | 2.17 (1.89-2.47) | 1.45 (1.30-1.61) | 2.55 (2.04-3.06) |
| **Women 75-84** | 1.58 (1.33-1.88) | 1.33 (1.20-1.47) | 2.36 (1.89-2.83) |
| **Women ≥85** | 1.38 (1.08-1.77) | 1.24 (1.13-1.37) | 2.36 (1.89-2.83) |

***Estimating the combined effects of risk factor change***

Deaths prevented/postponed in 2020 in the scenarios of risk factor change could be negative (deaths postponed through risk factor reduction) or positive (additional deaths caused by adverse trends). We assumed that changes across all six risk factors would have a cumulative rather than a merely additive effect on mortality. The summation of deaths avoided was therefore multiplied by a cumulative risk reduction factor which was estimated using the standard formula^6,10-12^:

*1-((1-a)×(1-b)×(1-c)×....×(1-n))*

where *a*, *b*, *c* and *n* denote the relative change in mortality attributable to a specific risk factor.

***Contribution of each risk factor to mortality gains***

Relative change in CHD mortality rates from an absolute change in continuous risk factors between 2007 and 2020 was calculated as:

*1-exponential(beta coefficient × absolute change in mean risk factor levels_2020-2007_)*

Relative change for dichotomous risk factors was calculated as:

*PARF × (ΔP_2020-2007_/P_2007_)*

Where P denotes prevalence in the baseline year (2007) and ΔP denotes the absolute change in prevalence between 2007 and 2020.

***Health Survey for England: response rates, missing data and risk factor definitions***

The Health Survey for England, an annual nationwide household survey of the English population, has been described in detail elsewhere.^13-15^ Briefly, members of a stratified random sample (drawn from the Postcode Address File) that is socio-demographically representative of the English population were invited to participate. Health Survey for England samples are selected using a multi-stage stratified probability design to give representative samples of the English non-institutional population at each survey year. Postcode sectors are selected at the first stage and household addresses are chosen randomly within the selected postcode sectors at the second stage. All adults within the selected households are eligible for interview. The annual household response rate was 75% in 2000, falling steadily to 66% in 2007. Data were collected at two visits: an interviewer’s visit, during which a questionnaire was administered, followed by a visit from a trained nurse for all those interviewed who agreed. The nurse visit among the general population sample includes measurements and collection of blood, as well as additional questioning including use of prescribed medications. [Visits from trained nurses took place in each survey year except 1999 and 2004]. For each risk factor the number of eligible participants at each relevant stage of the survey is shown in **Table C**. Reasons for missing data include refusals, don’t knows and ineligibility (e.g., participants with clotting and bleeding disorders, or respondents who ate, drank or smoked in the 30 minutes prior to the measurement of blood pressure). Survey data from 2003 onwards were weighted for non-response, with different weights applied to the main interview, nurse visit, and blood samples. Non-response weights were not produced for data prior to 2003 due to good response rates in earlier surveys. Risk factor definitions for the six risk factors used in our study are detailed in **Table C**.

**Table C. Risk factor definitions from the Health Survey for England and participants at each stage**

| **Risk factor** | **Health Survey for England years** | **Description** | **Sample size** |
| --- | --- | --- | --- |
| **Current cigarette smoking** | 1994-2008 (inclusive) | Respondents are asked about their smoking status within the face to face interview. The interview collected information about use of various tobacco products including cigarettes, cigars and, for men, pipes. | *Main interview*: 159,200 *Analysed*: 158,959  *Missing data*: 241 |
| **Systolic blood pressure (mmHg)** | 1994-2008 (except 1999 and 2004) | Three blood pressure readings were taken, at one-minute intervals, using an appropriately sized cuff on the right arm, with the informant in a seated position after five minutes’ rest. Calculated as the mean of the 2^nd^ and 3^rd^ readings for those who had not eaten, consumed alcohol or smoked in the 30 minutes prior to measurement. Those reporting taking blood pressure lowering drugs were included. Informants are excluded if they were pregnant. | *Main interview*: 146,277  *Nurse visit:* 118,906  *Analysed*: 104,314  *Missing data*:42,399 |
| **Body mass index (kg/m^2^)** | 1994-2008 (inclusive) | Height was measured using a portable stadiometer with a sliding head plate, a base plate and three connecting rods marked with a metric measuring scale. Informants were asked to remove shoes. One measurement was taken, with the informant stretching to the maximum height and the head positioned in the Frankfort plane. The reading was recorded to the nearest millimetre. Weight was measured using Soehnle, Seca and Tanita electronic scales with a digital display. Informants were asked to remove shoes and any bulky clothing. A single measurement was recorded to the nearest 100g. Informants who were pregnant, chair-bound, or unsteady on their feet were not weighed. Informants who weighed ≥ 130 kg were asked for their estimated weights because the scales are inaccurate above this level. These estimated weights were included in the analysis. Data from those who were considered by the interviewer to have unreliable measurements, for example those who had excessive clothing on, were excluded from the analysis.  BMI was measured as weight (kg) divided by height squared (m^2^) for all respondents with valid height and weight measurements. | *Main interview*: 159,200  *Analysed*: 140,577  *Missing data*: 18,623 |
| **Total cholesterol (mmol/l)** | 1994,1998,2003,2006,  2008 | Following written consent from eligible informants, three non-fasting blood samples (6 ml plain, 4 ml EDTA, and 4.5 ml citrate tubes) were collected by survey nurses.  Those reporting taking lipid lowering drugs were included. | *Main interview*: 67,081  *Nurse visit*: 53,493  *Blood sample*: 41,937  *Analysed*: 40,876  *Missing data*: 26,205 |
| **Diabetes** | 1994,1998,2003,2006 | Those reporting diabetes that was doctor-diagnosed, excluding women who had only had diabetes during pregnancy. | *Main interview*: 52,081  *Analysed*: 52,064  *Missing data*: 17 |
| **Physical activity** | 1998,2003,2006,2008 | Adults’ physical activity in the 4 weeks prior to interview is measured by examining overall participation; frequency of participation in activities that lasted ≥ 15 minutes; type of activities; and duration of activities. A question about intensity of the activity was asked for sports and exercise and for walking. High levels defined as spending 30 minutes or more of moderate or vigorous activity on at least five days per week. Occupational activity was excluded from estimation of summary activity levels. | *Main interview*: 51,631  *Analysed*: 51,545  *Missing data*: 86 |

# Table D. Risk factor levels in the Health Survey for England using pooled data (2003-08) by age-group, gender and deprivation quintile

| **Risk factor and age** |  |  | **Men** |  |  |  |  |  |  | **Women** |  |  |  |
| --- | --- | --- | --- | --- | --- | --- | --- | --- | --- | --- | --- | --- | --- |
|  | **England** | **Index of Multiple Deprivation 2007** | | | | |  | **England** | **Index of Multiple Deprivation 2007** | | | | |
|  |  | **Q1** | **Q2** | **Q3** | **Q4** | **Q5** |  |  | **Q1** | **Q2** | **Q3** | **Q4** | **Q5** |
| **Smoking (%): 2003 to 2008 (inclusive)** | | | | | | | | | | | | | |
| ***≥25*** | ***24.8*** | ***16.7*** | ***19.9*** | ***23.6*** | ***28.8*** | ***37.7*** |  | ***22.2*** | ***14.1*** | ***16.9*** | ***22.1*** | ***27.0*** | ***34.1*** |
| 25-34 | 35.3 | 27.2 | 30.4 | 32.7 | 39.6 | 42.8 |  | 26.5 | 19.2 | 20.1 | 27.0 | 28.9 | 33.6 |
| 35-44 | 29.5 | 19.1 | 23.6 | 28.3 | 34.7 | 43.8 |  | 26.6 | 15.9 | 19.5 | 26.1 | 34.6 | 38.7 |
| 45-54 | 24.3 | 15.9 | 18.1 | 23.8 | 25.9 | 43.9 |  | 24.1 | 14.1 | 18.4 | 24.8 | 27.8 | 42.3 |
| 55-64 | 19.6 | 11.6 | 14.0 | 19.9 | 25.9 | 34.3 |  | 19.4 | 12.6 | 14.2 | 18.5 | 25.6 | 34.3 |
| 65-74 | 13.1 | 7.5 | 12.1 | 11.1 | 16.6 | 21.2 |  | 13.5 | 8.3 | 11.1 | 12.9 | 17.5 | 21.6 |
| ≥75 | 7.7 | 4.4 | 5.7 | 7.4 | 9.9 | 15.2 |  | 8.5 | 5.0 | 8.0 | 7.9 | 10.7 | 12.4 |
| **Diabetes (%): 2003 and 2006** | | | | | | | | | | | | | |
| ***≥25*** | ***5.4*** | ***4.3*** | ***4.5*** | ***5.3*** | ***5.6*** | ***8.3*** |  | ***3.7*** | ***3.3*** | ***2.6*** | ***3.2*** | ***4.4*** | ***6.1*** |
| 25-34 | 0.7 | 0.3 | 1.0 | 0.5 | 0.4 | 1.3 |  | 1.0 | 2.1 | 0.8 | 0.7 | 1.0 | 0.8 |
| 35-44 | 2.6 | 2.5 | 2.2 | 1.1 | 3.5 | 3.6 |  | 1.3 | 1.0 | 1.0 | 1.7 | 0.8 | 2.6 |
| 45-54 | 4.7 | 2.6 | 3.3 | 6.0 | 4.0 | 9.0 |  | 3.0 | 2.3 | 1.6 | 2.8 | 3.3 | 6.3 |
| 55-64 | 8.3 | 8.4 | 4.9 | 8.1 | 8.5 | 13.3 |  | 5.3 | 5.0 | 3.8 | 4.2 | 6.6 | 8.8 |
| 65-74 | 13.6 | 10.5 | 13.4 | 11.3 | 15.9 | 20.2 |  | 9.3 | 7.1 | 6.6 | 7.6 | 15.2 | 12.6 |
| ≥75 | 11.6 | 8.6 | 11.8 | 15.9 | 9.9 | 11.5 |  | 9.7 | 8.3 | 8.1 | 8.9 | 8.4 | 17.5 |
| **Physical inactivity (%): 2006 and 2008** | | | | | | | | | | | | | |
| ***≥25*** | ***72.6*** | ***70.3*** | ***70.9*** | ***71.9*** | ***72.8*** | ***78.2*** |  | ***75.0*** | ***74.1*** | ***73.2*** | ***74.4*** | ***75.6*** | ***79.8*** |
| 25-34 | 62.1 | 61.7 | 61.8 | 61.1 | 60.0 | 65.7 |  | 68.8 | 72.7 | 67.4 | 69.4 | 66.3 | 68.9 |
| 35-44 | 68.6 | 64.3 | 66.2 | 68.6 | 67.9 | 75.9 |  | 70.8 | 70.4 | 68.5 | 70.3 | 71.6 | 73.4 |
| 45-54 | 74.0 | 70.4 | 70.5 | 76.0 | 76.0 | 79.1 |  | 74.1 | 68.7 | 70.2 | 74.9 | 77.1 | 83.8 |
| 55-64 | 77.4 | 74.7 | 75.5 | 78.4 | 75.9 | 84.7 |  | 76.6 | 74.2 | 76.7 | 73.1 | 77.2 | 85.3 |
| 65-74 | 81.1 | 81.3 | 81.8 | 72.8 | 83.6 | 87.5 |  | 84.0 | 84.0 | 81.9 | 81.8 | 86.3 | 87.9 |
| ≥75 | 90.8 | 90.3 | 89.3 | 86.8 | 96.7 | 93.3 |  | 95.0 | 93.8 | 94.9 | 95.0 | 94.8 | 96.8 |
| **Systolic blood pressure (mmHg): 2003 to 2008 (excluding 2004)** | | | | | | | | | | | | | |
| ***≥25*** | ***131.2*** | ***130.6*** | ***131.3*** | ***131.5*** | ***131.4*** | ***131.7*** |  | ***124.6*** | ***123.7*** | ***124.3*** | ***124.5*** | ***125.4*** | ***125.8*** |
| 25-34 | 126.5 | 126.1 | 128.4 | 126.2 | 126.0 | 126.4 |  | 114.3 | 114.0 | 114.7 | 114.3 | 114.8 | 113.6 |
| 35-44 | 127.6 | 127.2 | 127.6 | 128.4 | 128.1 | 126.6 |  | 117.4 | 116.9 | 117.0 | 117.3 | 117.8 | 118.1 |
| 45-54 | 130.7 | 130.0 | 130.2 | 130.9 | 130.9 | 132.1 |  | 124.5 | 122.7 | 123.9 | 124.4 | 126.3 | 126.2 |
| 55-64 | 135.1 | 134.2 | 134.8 | 135.2 | 135.7 | 136.2 |  | 131.4 | 129.6 | 130.1 | 132.3 | 132.9 | 134.0 |
| 65-74 | 137.8 | 136.5 | 137.2 | 139.0 | 138.9 | 137.7 |  | 138.8 | 138.3 | 140.1 | 137.9 | 137.4 | 141.1 |
| ≥75 | 139.8 | 139.4 | 139.1 | 139.0 | 139.7 | 143.2 |  | 143.1 | 143.4 | 142.0 | 141.9 | 144.3 | 144.2 |
| **Total cholesterol (mmol/l): 2003, 2006 and 2008** | | | | | | | | | | | | | |
| ***≥25*** | ***5.5*** | ***5.6*** | ***5.6*** | ***5.5*** | ***5.5*** | ***5.4*** |  | ***5.5*** | ***5.6*** | ***5.6*** | ***5.5*** | ***5.5*** | ***5.5*** |
| 25-34 | 5.2 | 5.2 | 5.2 | 5.2 | 5.2 | 5.2 |  | 4.9 | 4.9 | 4.9 | 5.0 | 4.8 | 4.9 |
| 35-44 | 5.7 | 5.7 | 5.7 | 5.7 | 5.7 | 5.5 |  | 5.2 | 5.2 | 5.2 | 5.1 | 5.3 | 5.3 |
| 45-54 | 5.8 | 5.8 | 5.9 | 5.8 | 5.7 | 5.7 |  | 5.7 | 5.7 | 5.7 | 5.8 | 5.7 | 5.8 |
| 55-64 | 5.7 | 5.7 | 5.7 | 5.7 | 5.6 | 5.5 |  | 6.1 | 6.2 | 6.2 | 6.1 | 5.9 | 6.0 |
| 65-74 | 5.3 | 5.4 | 5.3 | 5.4 | 5.1 | 5.1 |  | 6.0 | 6.1 | 6.0 | 6.0 | 5.9 | 5.9 |
| ≥75 | 5.0 | 5.0 | 5.1 | 5.0 | 4.8 | 5.1 |  | 5.8 | 5.8 | 5.8 | 5.8 | 5.8 | 5.6 |
| **Body mass index (kg/m^2^): 2003 to 2008 (inclusive)** | | | | | | | | | | | | | |
| ***≥25*** | ***27.7*** | ***27.5*** | ***27.8*** | ***27.7*** | ***27.8*** | ***27.5*** |  | ***27.2*** | ***26.5*** | ***26.7*** | ***27.2*** | ***27.6*** | ***28.1*** |
| 25-34 | 26.4 | 26.6 | 26.8 | 26.3 | 26.4 | 26.2 |  | 25.9 | 25.6 | 25.4 | 25.6 | 26.0 | 26.5 |
| 35-44 | 27.8 | 27.5 | 27.9 | 27.8 | 28.1 | 27.5 |  | 26.9 | 26.1 | 26.4 | 26.9 | 27.4 | 28.0 |
| 45-54 | 28.1 | 28.0 | 28.1 | 28.2 | 28.4 | 27.9 |  | 27.6 | 26.9 | 27.1 | 27.7 | 28.1 | 28.7 |
| 55-64 | 28.3 | 28.0 | 28.4 | 28.5 | 28.4 | 28.5 |  | 27.9 | 27.2 | 27.6 | 28.1 | 28.4 | 29.1 |
| 65-74 | 28.2 | 27.8 | 28.1 | 28.4 | 28.5 | 28.5 |  | 28.3 | 27.7 | 28.0 | 28.4 | 28.7 | 29.1 |
| ≥75 | 27.1 | 26.8 | 27.2 | 27.5 | 27.1 | 26.8 |  | 27.3 | 26.7 | 27.3 | 27.7 | 27.2 | 27.5 |

Pooled survey data was used in our modelling approach to estimate socioeconomic gradients - which were assumed unchanged from the baseline year to 2020 in the optimal, intermediate and worst case scenarios. Socioeconomic gradients computed as ratio of IMD quintile/England. Q1 = least deprived; Q5 = most deprived. Estimates for 25+ were age-standardised using the European Union (EU) standard population.

**Table E. Smoothed baseline (2007) risk factor levels by age-group, gender and deprivation quintile**

| **Risk factor and age** |  |  | **Men** |  |  |  |  |  |  | **Women** |  |  |  |
| --- | --- | --- | --- | --- | --- | --- | --- | --- | --- | --- | --- | --- | --- |
|  | **England** | **Index of Multiple Deprivation 2007** | | | | |  | **England** | **Index of Multiple Deprivation 2007** | | | | |
|  |  | **Q1** | **Q2** | **Q3** | **Q4** | **Q5** |  |  | **Q1** | **Q2** | **Q3** | **Q4** | **Q5** |
| **Smoking (%)** | | | | | | | | | | | | | |
| ***≥25*** | ***24.4*** | ***16.5*** | ***19.6*** | ***23.3*** | ***28.4*** | ***37.1*** |  | ***21.5*** | ***13.7*** | ***16.3*** | ***21.4*** | ***26.1*** | ***33.0*** |
| 25-34 | 34.9 | 26.9 | 30.1 | 32.3 | 39.2 | 42.3 |  | 25.4 | 18.4 | 19.3 | 25.9 | 27.7 | 32.3 |
| 35-44 | 29.3 | 19.0 | 23.5 | 28.1 | 34.4 | 43.6 |  | 25.8 | 15.4 | 19.0 | 25.4 | 33.6 | 37.6 |
| 45-54 | 23.8 | 15.6 | 17.8 | 23.4 | 25.4 | 43.0 |  | 23.3 | 13.7 | 17.9 | 24.0 | 26.9 | 41.0 |
| 55-64 | 19.4 | 11.5 | 13.9 | 19.6 | 25.6 | 33.9 |  | 18.6 | 12.0 | 13.5 | 17.6 | 24.4 | 32.8 |
| 65-74 | 12.5 | 7.2 | 11.6 | 10.6 | 15.8 | 20.2 |  | 13.5 | 8.4 | 11.2 | 12.9 | 17.6 | 21.7 |
| ≥75 | 7.4 | 4.2 | 5.4 | 7.0 | 9.5 | 14.5 |  | 8.0 | 4.7 | 7.5 | 7.4 | 10.0 | 11.6 |
| **Diabetes (%)** | | | | | | | | | | | | | |
| ***≥25*** | ***6.3*** | ***5.0*** | ***5.3*** | ***6.2*** | ***6.5*** | ***9.7*** |  | ***4.4*** | ***4.0*** | ***3.1*** | ***3.8*** | ***5.3*** | ***7.2*** |
| 25-34 | 0.8 | 0.4 | 1.1 | 0.6 | 0.4 | 1.5 |  | 1.4 | 2.8 | 1.0 | 1.0 | 1.3 | 1.0 |
| 35-44 | 3.1 | 3.0 | 2.6 | 1.3 | 4.2 | 4.2 |  | 1.4 | 1.0 | 1.1 | 1.8 | 0.8 | 2.8 |
| 45-54 | 5.8 | 3.1 | 4.0 | 7.3 | 5.0 | 11.0 |  | 3.6 | 2.7 | 2.0 | 3.3 | 3.9 | 7.5 |
| 55-64 | 8.7 | 8.9 | 5.2 | 8.5 | 9.0 | 14.0 |  | 6.4 | 6.0 | 4.6 | 5.0 | 8.0 | 10.6 |
| 65-74 | 17.1 | 13.1 | 16.8 | 14.2 | 19.9 | 25.3 |  | 11.0 | 8.4 | 7.9 | 9.0 | 18.0 | 14.9 |
| ≥75 | 13.3 | 9.8 | 13.5 | 18.1 | 11.3 | 13.2 |  | 11.3 | 9.6 | 9.4 | 10.3 | 9.7 | 20.2 |
| **Physical inactivity (%)** | | | | | | | | | | | | | |
| ***≥25*** | ***73.4*** | ***71.1*** | ***71.7*** | ***72.6*** | ***73.6*** | ***79.1*** |  | ***75.8*** | ***74.9*** | ***73.9*** | ***75.2*** | ***76.4*** | ***80.6*** |
| 25-34 | 64.0 | 63.6 | 63.7 | 63.0 | 61.8 | 67.6 |  | 70.4 | 74.4 | 69.0 | 71.1 | 67.9 | 70.6 |
| 35-44 | 69.2 | 64.9 | 66.8 | 69.2 | 68.5 | 76.6 |  | 71.7 | 71.3 | 69.4 | 71.2 | 72.5 | 74.4 |
| 45-54 | 74.3 | 70.7 | 70.8 | 76.3 | 76.3 | 79.5 |  | 74.6 | 69.1 | 70.7 | 75.4 | 77.6 | 84.3 |
| 55-64 | 77.8 | 75.1 | 75.9 | 78.9 | 76.3 | 85.1 |  | 77.0 | 74.6 | 77.1 | 73.4 | 77.5 | 85.7 |
| 65-74 | 81.8 | 82.0 | 82.5 | 73.5 | 84.4 | 88.3 |  | 84.5 | 84.5 | 82.4 | 82.3 | 86.8 | 88.4 |
| ≥75 | 91.0 | 90.5 | 89.5 | 86.9 | 96.9 | 93.5 |  | 95.1 | 93.9 | 95.1 | 95.2 | 95.0 | 96.9 |
| **Systolic blood pressure (mmHg)** | | | | | | | | | | | | | |
| ***≥25*** | ***130.8*** | ***130.1*** | ***130.9*** | ***131.0*** | ***131.0*** | ***131.2*** |  | ***124.0*** | ***123.1*** | ***123.7*** | ***123.9*** | ***124.7*** | ***125.1*** |
| 25-34 | 126.4 | 125.9 | 128.3 | 126.1 | 125.8 | 126.2 |  | 113.9 | 113.6 | 114.3 | 113.9 | 114.4 | 113.2 |
| 35-44 | 127.3 | 127.0 | 127.3 | 128.1 | 127.8 | 126.3 |  | 117.0 | 116.5 | 116.7 | 116.9 | 117.5 | 117.7 |
| 45-54 | 130.3 | 129.7 | 129.9 | 130.6 | 130.5 | 131.7 |  | 123.8 | 122.0 | 123.2 | 123.7 | 125.6 | 125.5 |
| 55-64 | 134.5 | 133.7 | 134.3 | 134.6 | 135.1 | 135.7 |  | 130.7 | 128.9 | 129.4 | 131.5 | 132.1 | 133.2 |
| 65-74 | 136.8 | 135.6 | 136.3 | 138.1 | 137.9 | 136.8 |  | 137.7 | 137.2 | 139.0 | 136.8 | 136.3 | 140.0 |
| ≥75 | 138.5 | 138.1 | 137.8 | 137.7 | 138.5 | 141.9 |  | 141.6 | 142.0 | 140.6 | 140.5 | 142.9 | 142.7 |
| **Total cholesterol (mmol/l)** | | | | | | | | | | | | | |
| ***≥25*** | ***5.5*** | ***5.5*** | ***5.5*** | ***5.5*** | ***5.4*** | ***5.4*** |  | ***5.5*** | ***5.5*** | ***5.5*** | ***5.5*** | ***5.5*** | ***5.5*** |
| 25-34 | 5.2 | 5.2 | 5.2 | 5.1 | 5.2 | 5.2 |  | 4.9 | 4.9 | 4.9 | 5.0 | 4.8 | 4.9 |
| 35-44 | 5.7 | 5.7 | 5.7 | 5.7 | 5.7 | 5.5 |  | 5.2 | 5.2 | 5.2 | 5.1 | 5.3 | 5.3 |
| 45-54 | 5.8 | 5.8 | 5.9 | 5.8 | 5.7 | 5.7 |  | 5.7 | 5.7 | 5.7 | 5.7 | 5.7 | 5.8 |
| 55-64 | 5.6 | 5.7 | 5.7 | 5.7 | 5.6 | 5.4 |  | 6.1 | 6.1 | 6.2 | 6.1 | 5.9 | 5.9 |
| 65-74 | 5.2 | 5.3 | 5.2 | 5.3 | 5.1 | 5.0 |  | 5.9 | 5.9 | 5.9 | 5.9 | 5.8 | 5.8 |
| ≥75 | 4.9 | 4.9 | 4.9 | 4.9 | 4.7 | 4.9 |  | 5.6 | 5.7 | 5.7 | 5.7 | 5.6 | 5.5 |
| **Body mass index (kg/m^2^)** | | | | | | | | | | | | | |
| ***≥25*** | ***27.8*** | ***27.6*** | ***27.9*** | ***27.8*** | ***28.0*** | ***27.7*** |  | ***27.3*** | ***26.7*** | ***26.9*** | ***27.3*** | ***27.7*** | ***28.2*** |
| 25-34 | 26.6 | 26.7 | 27.0 | 26.4 | 26.5 | 26.3 |  | 26.0 | 25.6 | 25.5 | 25.7 | 26.1 | 26.6 |
| 35-44 | 27.9 | 27.6 | 28.0 | 27.9 | 28.2 | 27.6 |  | 27.0 | 26.2 | 26.5 | 27.0 | 27.5 | 28.2 |
| 45-54 | 28.2 | 28.0 | 28.2 | 28.3 | 28.5 | 28.0 |  | 27.7 | 26.9 | 27.1 | 27.8 | 28.2 | 28.8 |
| 55-64 | 28.5 | 28.1 | 28.5 | 28.6 | 28.6 | 28.7 |  | 28.0 | 27.3 | 27.7 | 28.2 | 28.5 | 29.2 |
| 65-74 | 28.5 | 28.0 | 28.3 | 28.7 | 28.8 | 28.7 |  | 28.4 | 27.9 | 28.1 | 28.5 | 28.9 | 29.3 |
| ≥75 | 27.2 | 26.9 | 27.3 | 27.6 | 27.2 | 26.9 |  | 27.4 | 26.8 | 27.5 | 27.8 | 27.3 | 27.7 |

Q1 = least deprived; Q5 = most deprived. Estimates for 25+ were age-standardised using the European Union (EU) standard population. Baseline values for IMD quintiles were **smoothed**. Smoothed values were obtained by multiplying national value in 2007 (estimated by fractional polynomial functions) - by the socioeconomic gradient (see **Table D**).

**Table F. Worst-case scenario: risk factor levels by age-group, gender and deprivation quintile**

| **Risk factor and age** |  |  | **Men** |  |  |  |  |  |  | **Women** |  |  |  |
| --- | --- | --- | --- | --- | --- | --- | --- | --- | --- | --- | --- | --- | --- |
|  | **England** | **Index of Multiple Deprivation 2007** | | | | |  | **England** | **Index of Multiple Deprivation 2007** | | | | |
|  |  | **Q1** | **Q2** | **Q3** | **Q4** | **Q5** |  |  | **Q1** | **Q2** | **Q3** | **Q4** | **Q5** |
| **Smoking (%)** | | | | | | | | | | | | | |
| ***≥25*** | ***24.4*** | ***16.5*** | ***19.6*** | ***23.3*** | ***28.4*** | ***37.1*** |  | ***21.5*** | ***13.7*** | ***16.3*** | ***21.4*** | ***26.1*** | ***33.0*** |
| 25-34 | 34.9 | 26.9 | 30.1 | 32.3 | 39.2 | 42.3 |  | 25.4 | 18.4 | 19.3 | 25.9 | 27.7 | 32.3 |
| 35-44 | 29.3 | 19.0 | 23.5 | 28.1 | 34.4 | 43.6 |  | 25.8 | 15.4 | 19.0 | 25.4 | 33.6 | 37.6 |
| 45-54 | 23.8 | 15.6 | 17.8 | 23.4 | 25.4 | 43.0 |  | 23.3 | 13.7 | 17.9 | 24.0 | 26.9 | 41.0 |
| 55-64 | 19.4 | 11.5 | 13.9 | 19.6 | 25.6 | 33.9 |  | 18.6 | 12.0 | 13.5 | 17.6 | 24.4 | 32.8 |
| 65-74 | 12.5 | 7.2 | 11.6 | 10.6 | 15.8 | 20.2 |  | 13.5 | 8.4 | 11.2 | 12.9 | 17.6 | 21.7 |
| ≥75 | 7.4 | 4.2 | 5.4 | 7.0 | 9.5 | 14.5 |  | 8.0 | 4.7 | 7.5 | 7.4 | 10.0 | 11.6 |
| **Diabetes (%)** | | | | | | | | | | | | | |
| ***≥25*** | ***17.6*** | ***14.1*** | ***14.7*** | ***17.3*** | ***18.2*** | ***27.0*** |  | ***12.4*** | ***11.0*** | ***8.8*** | ***10.7*** | ***14.7*** | ***20.2*** |
| 25-34 | 2.4 | 1.1 | 3.4 | 1.7 | 1.2 | 4.4 |  | 3.4 | 7.0 | 2.6 | 2.4 | 3.2 | 2.6 |
| 35-44 | 8.5 | 8.3 | 7.3 | 3.7 | 11.5 | 11.7 |  | 4.5 | 3.2 | 3.3 | 5.6 | 2.5 | 8.6 |
| 45-54 | 15.4 | 8.3 | 10.7 | 19.5 | 13.2 | 29.4 |  | 10.1 | 7.5 | 5.5 | 9.1 | 10.9 | 20.8 |
| 55-64 | 27.0 | 27.6 | 16.1 | 26.3 | 27.7 | 43.4 |  | 17.7 | 16.6 | 12.7 | 13.8 | 22.0 | 29.2 |
| 65-74 | 44.6 | 34.4 | 44.0 | 37.0 | 52.0 | 66.1 |  | 30.9 | 23.7 | 22.1 | 25.2 | 50.6 | 41.9 |
| ≥75 | 38.0 | 28.2 | 38.6 | 51.8 | 32.4 | 37.7 |  | 32.3 | 27.4 | 26.8 | 29.5 | 27.8 | 57.9 |
| **Physical inactivity (%)** | | | | | | | | | | | | | |
| ***≥25*** | ***73.4*** | ***71.1*** | ***71.7*** | ***72.6*** | ***73.6*** | ***79.1*** |  | ***75.8*** | ***74.9*** | ***73.9*** | ***75.2*** | ***76.4*** | ***80.6*** |
| 25-34 | 64.0 | 63.6 | 63.7 | 63.0 | 61.8 | 67.6 |  | 70.4 | 74.4 | 69.0 | 71.1 | 67.9 | 70.6 |
| 35-44 | 69.2 | 64.9 | 66.8 | 69.2 | 68.5 | 76.6 |  | 71.7 | 71.3 | 69.4 | 71.2 | 72.5 | 74.4 |
| 45-54 | 74.3 | 70.7 | 70.8 | 76.3 | 76.3 | 79.5 |  | 74.6 | 69.1 | 70.7 | 75.4 | 77.6 | 84.3 |
| 55-64 | 77.8 | 75.1 | 75.9 | 78.9 | 76.3 | 85.1 |  | 77.0 | 74.6 | 77.1 | 73.4 | 77.5 | 85.7 |
| 65-74 | 81.8 | 82.0 | 82.5 | 73.5 | 84.4 | 88.3 |  | 84.5 | 84.5 | 82.4 | 82.3 | 86.8 | 88.4 |
| ≥75 | 91.0 | 90.5 | 89.5 | 86.9 | 96.9 | 93.5 |  | 95.1 | 93.9 | 95.1 | 95.2 | 95.0 | 96.9 |
| **Systolic blood pressure (mmHg)** | | | | | | | | | | | | | |
| ***≥25*** | ***130.8*** | ***130.1*** | ***130.9*** | ***131.0*** | ***131.0*** | ***131.2*** |  | ***124.0*** | ***123.1*** | ***123.7*** | ***123.9*** | ***124.7*** | ***125.1*** |
| 25-34 | 126.4 | 125.9 | 128.3 | 126.1 | 125.8 | 126.2 |  | 113.9 | 113.6 | 114.3 | 113.9 | 114.4 | 113.2 |
| 35-44 | 127.3 | 127.0 | 127.3 | 128.1 | 127.8 | 126.3 |  | 117.0 | 116.5 | 116.7 | 116.9 | 117.5 | 117.7 |
| 45-54 | 130.3 | 129.7 | 129.9 | 130.6 | 130.5 | 131.7 |  | 123.8 | 122.0 | 123.2 | 123.7 | 125.6 | 125.5 |
| 55-64 | 134.5 | 133.7 | 134.3 | 134.6 | 135.1 | 135.7 |  | 130.7 | 128.9 | 129.4 | 131.5 | 132.1 | 133.2 |
| 65-74 | 136.8 | 135.6 | 136.3 | 138.1 | 137.9 | 136.8 |  | 137.7 | 137.2 | 139.0 | 136.8 | 136.3 | 140.0 |
| ≥75 | 138.5 | 138.1 | 137.8 | 137.7 | 138.5 | 141.9 |  | 141.6 | 142.0 | 140.6 | 140.5 | 142.9 | 142.7 |
| **Total cholesterol (mmol/l)** | | | | | | | | | | | | | |
| ***≥25*** | ***5.5*** | ***5.5*** | ***5.5*** | ***5.5*** | ***5.4*** | ***5.4*** |  | ***5.5*** | ***5.5*** | ***5.5*** | ***5.5*** | ***5.5*** | ***5.5*** |
| 25-34 | 5.2 | 5.2 | 5.2 | 5.1 | 5.2 | 5.2 |  | 4.9 | 4.9 | 4.9 | 5.0 | 4.8 | 4.9 |
| 35-44 | 5.7 | 5.7 | 5.7 | 5.7 | 5.7 | 5.5 |  | 5.2 | 5.2 | 5.2 | 5.1 | 5.3 | 5.3 |
| 45-54 | 5.8 | 5.8 | 5.9 | 5.8 | 5.7 | 5.7 |  | 5.7 | 5.7 | 5.7 | 5.7 | 5.7 | 5.8 |
| 55-64 | 5.6 | 5.7 | 5.7 | 5.7 | 5.6 | 5.4 |  | 6.1 | 6.1 | 6.2 | 6.1 | 5.9 | 5.9 |
| 65-74 | 5.2 | 5.3 | 5.2 | 5.3 | 5.1 | 5.0 |  | 5.9 | 5.9 | 5.9 | 5.9 | 5.8 | 5.8 |
| ≥75 | 4.9 | 4.9 | 4.9 | 4.9 | 4.7 | 4.9 |  | 5.6 | 5.7 | 5.7 | 5.7 | 5.6 | 5.5 |
| **Body mass index (kg/m^2^)** | | | | | | | | | | | | | |
| ***≥25*** | ***29.6*** | ***29.4*** | ***29.8*** | ***29.7*** | ***29.8*** | ***29.5*** |  | ***29.1*** | ***28.4*** | ***28.7*** | ***29.1*** | ***29.5*** | ***30.1*** |
| 25-34 | 28.3 | 28.5 | 28.8 | 28.2 | 28.3 | 28.1 |  | 27.7 | 27.4 | 27.2 | 27.5 | 27.9 | 28.3 |
| 35-44 | 29.7 | 29.4 | 29.9 | 29.7 | 30.1 | 29.4 |  | 28.8 | 28.0 | 28.3 | 28.8 | 29.4 | 30.0 |
| 45-54 | 30.1 | 29.9 | 30.1 | 30.2 | 30.5 | 29.9 |  | 29.5 | 28.7 | 29.0 | 29.6 | 30.1 | 30.7 |
| 55-64 | 30.4 | 30.0 | 30.5 | 30.5 | 30.5 | 30.6 |  | 29.9 | 29.1 | 29.5 | 30.1 | 30.4 | 31.2 |
| 65-74 | 30.4 | 29.9 | 30.2 | 30.6 | 30.7 | 30.7 |  | 30.3 | 29.7 | 30.0 | 30.4 | 30.8 | 31.2 |
| ≥75 | 29.0 | 28.7 | 29.1 | 29.4 | 29.1 | 28.7 |  | 29.2 | 28.6 | 29.3 | 29.7 | 29.1 | 29.5 |

Q1 = least deprived; Q5 = most deprived. Estimates for 25+ were age-standardised using the European Union (EU) standard population. Smoking, physical activity, SBP and total cholesterol levels assumed to remain unchanged. Diabetes levels assumed to reach 15% in England in 2020, with age, gender and socioeconomic gradients held fixed at 2007 levels. Annual increase in BMI was set at 0.5%, similar to the increases observed in the US over 1990-2005.^16^

**Table G. Assuming current trends continue: risk factor levels by age-group, gender and deprivation quintile**

| **Risk factor and age** |  |  | **Men** |  |  |  |  |  |  | **Women** |  |  |  |
| --- | --- | --- | --- | --- | --- | --- | --- | --- | --- | --- | --- | --- | --- |
|  | **England** | **Q1** | **Q2** | **Q3** | **Q4** | **Q5** |  | **England** | **Q1** | **Q2** | **Q3** | **Q4** | **Q5** |
| **Smoking (%)** | | | | | | | | | | | | | |
| ***≥25*** | ***20.7*** | ***13.3*** | ***16.5*** | ***19.4*** | ***25.5*** | ***32.7*** |  | ***12.9*** | ***7.4*** | ***12.8*** | ***18.7*** | ***22.7*** | ***27.8*** |
| 25-34 | 32.0 | 25.5 | 29.6 | 28.3 | 37.2 | 33.5 |  | 11.8 | 2.4 | 15.9 | 24.8 | 20.8 | 23.0 |
| 35-44 | 26.7 | 16.0 | 22.0 | 23.9 | 32.4 | 37.5 |  | 12.0 | 12.3 | 16.2 | 25.6 | 36.9 | 30.3 |
| 45-54 | 18.7 | 10.5 | 11.1 | 19.8 | 17.2 | 42.2 |  | 18.3 | 9.1 | 12.9 | 19.7 | 18.9 | 41.1 |
| 55-64 | 15.4 | 7.7 | 9.9 | 16.1 | 22.6 | 34.2 |  | 13.7 | 8.9 | 9.9 | 11.5 | 22.7 | 27.7 |
| 65-74 | 7.8 | 4.8 | 7.6 | 5.4 | 18.2 | 13.2 |  | 9.1 | 4.9 | 8.2 | 8.9 | 14.1 | 16.2 |
| ≥75 | 4.4 | 1.7 | 3.5 | 3.7 | 9.4 | 9.4 |  | 6.0 | 2.3 | 6.2 | 5.9 | 8.3 | 10.0 |
| **Diabetes (%)** | | | | | | | | | | | | | |
| ***≥25*** | ***11.7*** | ***8.8*** | ***8.9*** | ***16.2*** | ***9.2*** | ***19.8*** |  | ***10.8*** | ***9.7*** | ***6.2*** | ***10.3*** | ***18.6*** | ***16.5*** |
| 25-34 | 0.9 | 0.4 | 1.4 | 1.0 | 0 | 5.2 |  | 4.8 | 5.0^a^ | 3.0 | 3.6 | 1.8 | 2.5 |
| 35-44 | 8.1 | 11.5 | 7.1 | 1.0 | 5.0^a^ | 6.1 |  | 2.4 | 0.4 | 3.4 | 5.0 | 3.9 | 4.1 |
| 45-54 | 15.0 | 2.9 | 9.6 | 38.3 | 10.6 | 35.6 |  | 9.9 | 6.7 | 1.6 | 7.8 | 27.8 | 25.8 |
| 55-64 | 12.9 | 16.3 | 7.2 | 14.4 | 10.3 | 23.2 |  | 16.8 | 20.2 | 12.0 | 13.6 | 27.1 | 24.0 |
| 65-74 | 25.0^a^ | 19.9 | 25.0^a^ | 28.1 | 30.0^a^ | 30.0^a^ |  | 24.4 | 25.2 | 13.1 | 23.8 | 52.8 | 31.4 |
| ≥75 | 24.3 | 9.9 | 15.8 | 30.0^a^ | 12.1 | 36.8 |  | 24.4 | 13.7 | 15.6 | 27.9 | 12.9 | 30.0^a^ |
| **Physical inactivity (%)** | | | | | | | | | | | | | |
| ***≥25*** | ***63.7*** | ***60.7*** | ***63.1*** | ***61.8*** | ***64.0*** | ***72.1*** |  | ***67.9*** | ***66.0*** | ***64.7*** | ***65.7*** | ***69.9*** | ***76.7*** |
| 25-34 | 53.0 | 54.1 | 56.3 | 51.3 | 45.6 | 59.6 |  | 61.9 | 69.1 | 61.0 | 61.2 | 53.9 | 65.7 |
| 35-44 | 57.6 | 51.2 | 55.5 | 58.0 | 54.6 | 69.7 |  | 64.9 | 62.8 | 57.9 | 65.3 | 71.8 | 67.7 |
| 45-54 | 62.9 | 56.6 | 59.8 | 65.1 | 68.9 | 68.8 |  | 65.9 | 55.8 | 61.2 | 65.7 | 72.2 | 78.2 |
| 55-64 | 68.3 | 65.2 | 65.4 | 68.5 | 67.3 | 79.8 |  | 66.4 | 60.9 | 66.7 | 58.7 | 69.6 | 85.0 |
| 65-74 | 77.5 | 77.7 | 83.9 | 61.5 | 84.3 | 84.1 |  | 77.9 | 78.7 | 72.6 | 73.1 | 81.2 | 88.1 |
| ≥75 | 88.3 | 89.1 | 83.2 | 82.7 | 99.0 | 93.0 |  | 93.2 | 93.4 | 93.9 | 89.6 | 92.8 | 98.1 |
| **Systolic blood pressure (mm Hg)** | | | | | | | | | | | | | |
| ***≥25*** | ***126.6*** | ***125.3*** | ***127.1*** | ***126.9*** | ***127.0*** | ***126.0*** |  | ***114.0*** | ***114.6*** | ***116.1*** | ***115.8*** | ***117.8*** | ***118.6*** |
| 25-34 | 125.7 | 122.3 | 127.5 | 122.7 | 123.3 | 123.2 |  | 107.1 | 105.5 | 109.0 | 106.8 | 108.5 | 105.7 |
| 35-44 | 125.1 | 126.9 | 124.5 | 126.9 | 126.4 | 123.0 |  | 111.2 | 111.0 | 109.9 | 110.1 | 112.1 | 112.8 |
| 45-54 | 127.0 | 125.4 | 126.8 | 128.1 | 127.0 | 128.6 |  | 108.2 | 114.3 | 117.9 | 118.8 | 122.2 | 121.2 |
| 55-64 | 128.4 | 126.4 | 129.6 | 128.6 | 130.5 | 128.5 |  | 121.2 | 118.0 | 119.6 | 122.8 | 122.2 | 127.6 |
| 65-74 | 127.2 | 125.5 | 127.6 | 130.3 | 129.0 | 124.0 |  | 126.2 | 126.7 | 128.5 | 122.5 | 125.1 | 130.6 |
| ≥75 | 128.1 | 127.2 | 127.9 | 126.2 | 128.7 | 133.6 |  | 127.8 | 129.8 | 125.2 | 125.9 | 129.7 | 129.4 |
| **Total cholesterol (mmol/l)** | | | | | | | | | | | | | |
| ***≥25*** | ***5.3*** | ***5.4*** | ***5.2*** | ***5.1*** | ***5.1*** | ***4.8*** |  | ***5.2*** | ***5.3*** | ***5.3*** | ***5.1*** | ***5.3*** | ***5.1*** |
| 25-34 | 5.2 | 5.1 | 5.0 | 4.9 | 5.0 | 5.1 |  | 4.9 | 4.7 | 4.7 | 4.9 | 4.8 | 4.4 |
| 35-44 | 5.7 | 5.6 | 5.6 | 5.3 | 5.7 | 5.0 |  | 5.2 | 4.9 | 5.2 | 4.8 | 5.2 | 5.1 |
| 45-54 | 5.8 | 5.8 | 5.8 | 5.8 | 5.2 | 4.9 |  | 5.7 | 5.7 | 5.7 | 5.8 | 5.7 | 5.8 |
| 55-64 | 5.5 | 5.7 | 5.2 | 5.1 | 5.1 | 5.2 |  | 5.2 | 6.2 | 6.2 | 5.1 | 5.8 | 5.7 |
| 65-74 | 4.7 | 5.1 | 4.7 | 4.5 | 4.3 | 3.9 |  | 5.3 | 5.8 | 4.6 | 4.7 | 5.2 | 5.1 |
| ≥75 | 3.8 | 3.8 | 4.0 | 4.0 | 4.1 | 3.7 |  | 4.6 | 4.7 | 4.6 | 4.7 | 4.3 | 4.3 |
| **Body mass index (kg/m^2^)** | | | | | | | | | | | | | |
| ***≥25*** | ***28.6*** | ***28.7*** | ***29.5*** | ***29.0*** | ***29.0*** | ***28.7*** |  | ***27.9*** | ***27.7*** | ***27.6*** | ***28.1*** | ***29.1*** | ***29.4*** |
| 25-34 | 26.9 | 27.9 | 28.3 | 26.8 | 26.5 | 27.5 |  | 26.3 | 27.0 | 27.1 | 26.8 | 27.3 | 27.5 |
| 35-44 | 28.7 | 28.9 | 29.9 | 29.3 | 29.9 | 28.1 |  | 27.8 | 27.6 | 27.5 | 27.3 | 29.3 | 29.6 |
| 45-54 | 28.9 | 29.2 | 29.8 | 29.4 | 30.0 | 29.0 |  | 28.0 | 27.7 | 27.3 | 28.7 | 30.4 | 30.5 |
| 55-64 | 29.8 | 29.1 | 30.0 | 29.9 | 30.2 | 29.7 |  | 28.5 | 27.8 | 27.8 | 28.7 | 29.1 | 29.3 |
| 65-74 | 30.1 | 29.2 | 29.8 | 30.3 | 28.9 | 30.9 |  | 29.6 | 29.0 | 28.6 | 29.6 | 30.7 | 30.8 |
| ≥75 | 28.2 | 27.6 | 28.9 | 28.7 | 27.6 | 27.8 |  | 28.7 | 28.3 | 28.7 | 29.4 | 28.1 | 29.2 |

Q1 = least deprived; Q5 = most deprived. Risk factor levels in 2020 estimated using fractional polynomial functions. Estimates for 25+ were age-standardised using the European Union (EU) standard population.

^a^ Indicates ceiling set on trends due to potential implausible values (especially for risk factors only covered in intermittent years). Upper age band set to 75+.

**Table H. Intermediate scenario (halfway between current and optimal): risk factor levels by age-group, gender and deprivation quintile**

| **Risk factor and age** |  |  |  | **Men** |  |  |  |  |  |  | **Women** |  |  |
| --- | --- | --- | --- | --- | --- | --- | --- | --- | --- | --- | --- | --- | --- |
|  | **England** | **Q1** | **Q2** | **Q3** | **Q4** | **Q5** |  | **England** | **Q1** | **Q2** | **Q3** | **Q4** | **Q5** |
| **Smoking (%)** | | | | | | | | | | | | | |
| ***≥25*** | ***17.2*** | ***11.6*** | ***13.8*** | ***16.4*** | ***20.0*** | ***26.2*** |  | ***15.7*** | ***10.0*** | ***11.9*** | ***15.7*** | ***19.1*** | ***24.2*** |
| 25-34 | 24.6 | 18.9 | 21.2 | 22.7 | 27.6 | 29.8 |  | 18.7 | 13.5 | 14.2 | 19.0 | 20.4 | 23.7 |
| 35-44 | 20.6 | 13.3 | 16.5 | 19.8 | 24.2 | 30.6 |  | 18.9 | 11.3 | 13.9 | 18.6 | 24.6 | 27.5 |
| 45-54 | 16.8 | 11.0 | 12.5 | 16.5 | 17.9 | 30.3 |  | 17.1 | 10.0 | 13.1 | 17.6 | 19.7 | 30.0 |
| 55-64 | 13.7 | 8.1 | 9.8 | 13.8 | 18.0 | 23.9 |  | 13.7 | 8.8 | 10.0 | 13.0 | 18.0 | 24.1 |
| 65-74 | 8.9 | 5.1 | 8.2 | 7.5 | 11.3 | 14.4 |  | 9.8 | 6.0 | 8.1 | 9.4 | 12.7 | 15.7 |
| ≥75 | 5.3 | 3.0 | 3.8 | 5.0 | 6.8 | 10.3 |  | 5.9 | 3.4 | 5.5 | 5.5 | 7.4 | 8.6 |
| **Diabetes (%)** | | | | | | | | | | | | | |
| ***≥25*** | ***5.5*** | ***4.1*** | ***4.3*** | ***5.0*** | ***5.3*** | ***7.9*** |  | ***3.9*** | ***3.8*** | ***3.0*** | ***3.6*** | ***5.0*** | ***6.9*** |
| 25-34 | 0.7 | 0.3 | 0.9 | 0.5 | 0.3 | 1.2 |  | 1.1 | 2.5 | 0.9 | 0.9 | 1.1 | 0.9 |
| 35-44 | 2.7 | 2.4 | 2.1 | 1.1 | 3.4 | 3.4 |  | 1.3 | 1.0 | 1.1 | 1.8 | 0.8 | 2.8 |
| 45-54 | 4.9 | 2.5 | 3.2 | 5.9 | 4.0 | 8.9 |  | 3.2 | 2.6 | 1.9 | 3.1 | 3.7 | 7.1 |
| 55-64 | 8.0 | 7.6 | 4.4 | 7.2 | 7.6 | 11.9 |  | 5.6 | 5.7 | 4.4 | 4.7 | 7.5 | 10.0 |
| 65-74 | 14.5 | 10.5 | 13.4 | 11.3 | 15.9 | 20.1 |  | 9.6 | 8.1 | 7.5 | 8.5 | 17.2 | 14.2 |
| ≥75 | 11.7 | 8.1 | 11.1 | 14.9 | 9.3 | 10.9 |  | 10.0 | 9.2 | 9.0 | 9.9 | 9.4 | 19.5 |
| **Physical inactivity (%)** | | | | | | | | | | | | | |
| ***≥25*** | ***51.7*** | ***50.1*** | ***50.5*** | ***51.2*** | ***51.8*** | ***55.7*** |  | ***52.9*** | ***52.3*** | ***51.6*** | ***52.5*** | ***53.3*** | ***56.3*** |
| 25-34 | 44.8 | 44.6 | 44.6 | 44.1 | 43.3 | 47.4 |  | 49.0 | 51.7 | 48.0 | 49.4 | 47.2 | 49.1 |
| 35-44 | 48.8 | 45.7 | 47.1 | 48.8 | 48.3 | 54.0 |  | 50.0 | 49.7 | 48.4 | 49.7 | 50.6 | 51.8 |
| 45-54 | 52.4 | 49.9 | 50.0 | 53.9 | 53.8 | 56.1 |  | 52.1 | 48.3 | 49.4 | 52.7 | 54.2 | 58.9 |
| 55-64 | 54.9 | 53.0 | 53.6 | 55.6 | 53.8 | 60.1 |  | 53.8 | 52.1 | 53.9 | 51.3 | 54.2 | 59.9 |
| 65-74 | 57.6 | 57.8 | 58.2 | 51.8 | 59.5 | 62.2 |  | 59.0 | 59.0 | 57.6 | 57.5 | 60.6 | 61.8 |
| ≥75 | 64.3 | 63.9 | 63.2 | 61.4 | 68.4 | 66.0 |  | 66.6 | 65.7 | 66.5 | 66.6 | 66.4 | 67.8 |
| **Systolic blood pressure (mm Hg)** | | | | | | | | | | | | | |
| ***≥25*** | ***125.8*** | ***125.2*** | ***125.9*** | ***126.0*** | ***126.0*** | ***126.2*** |  | ***119.0*** | ***118.1*** | ***118.7*** | ***118.9*** | ***119.7*** | ***120.1*** |
| 25-34 | 121.4 | 120.9 | 123.2 | 121.1 | 120.8 | 121.2 |  | 108.9 | 108.6 | 109.3 | 108.9 | 109.4 | 108.3 |
| 35-44 | 122.3 | 122.0 | 122.3 | 123.1 | 122.8 | 121.4 |  | 112.0 | 111.6 | 111.7 | 111.9 | 112.4 | 112.7 |
| 45-54 | 125.3 | 124.7 | 124.9 | 125.6 | 125.5 | 126.7 |  | 118.8 | 117.1 | 118.2 | 118.7 | 120.5 | 120.4 |
| 55-64 | 129.5 | 128.7 | 129.3 | 129.6 | 130.1 | 130.6 |  | 125.7 | 123.9 | 124.5 | 126.5 | 127.1 | 128.1 |
| 65-74 | 131.8 | 130.7 | 131.3 | 133.0 | 132.9 | 131.8 |  | 132.7 | 132.2 | 134.0 | 131.8 | 131.4 | 134.9 |
| ≥75 | 133.5 | 133.1 | 132.9 | 132.8 | 133.5 | 136.8 |  | 136.6 | 137.0 | 135.6 | 135.6 | 137.8 | 137.7 |
| **Total cholesterol (mmol/l)** | | | | | | | | | | | | | |
| ***≥25*** | ***5.2*** | ***5.2*** | ***5.2*** | ***5.2*** | ***5.1*** | ***5.1*** |  | ***5.2*** | ***5.2*** | ***5.2*** | ***5.2*** | ***5.2*** | ***5.2*** |
| 25-34 | 4.9 | 4.9 | 4.9 | 4.8 | 4.9 | 4.9 |  | 4.6 | 4.6 | 4.6 | 4.6 | 4.5 | 4.6 |
| 35-44 | 5.4 | 5.4 | 5.4 | 5.4 | 5.4 | 5.2 |  | 4.9 | 4.9 | 4.9 | 4.8 | 5.0 | 5.0 |
| 45-54 | 5.5 | 5.5 | 5.6 | 5.5 | 5.4 | 5.4 |  | 5.4 | 5.4 | 5.4 | 5.4 | 5.4 | 5.5 |
| 55-64 | 5.3 | 5.4 | 5.4 | 5.4 | 5.3 | 5.1 |  | 5.8 | 5.8 | 5.9 | 5.8 | 5.6 | 5.6 |
| 65-74 | 4.9 | 5.0 | 4.9 | 5.0 | 4.8 | 4.7 |  | 5.6 | 5.6 | 5.6 | 5.6 | 5.5 | 5.5 |
| ≥75 | 4.6 | 4.6 | 4.6 | 4.6 | 4.4 | 4.6 |  | 5.3 | 5.4 | 5.4 | 5.4 | 5.3 | 5.2 |
| **Body mass index (kg/m^2^)** | | | | | | | | | | | | | |
| ***≥25*** | ***26.8*** | ***26.7*** | ***27.0*** | ***26.9*** | ***27.0*** | ***26.7*** |  | ***25.7*** | ***25.1*** | ***25.3*** | ***25.7*** | ***26.1*** | ***26.6*** |
| 25-34 | 25.7 | 25.8 | 26.0 | 25.5 | 25.6 | 25.4 |  | 24.4 | 24.2 | 24.0 | 24.2 | 24.6 | 25.0 |
| 35-44 | 26.9 | 26.7 | 27.1 | 26.9 | 27.3 | 26.7 |  | 25.4 | 24.7 | 24.9 | 25.4 | 25.9 | 26.5 |
| 45-54 | 27.3 | 27.1 | 27.2 | 27.4 | 27.6 | 27.1 |  | 26.1 | 25.4 | 25.6 | 26.1 | 26.6 | 27.1 |
| 55-64 | 27.5 | 27.2 | 27.6 | 27.6 | 27.6 | 27.7 |  | 26.4 | 25.7 | 26.1 | 26.6 | 26.8 | 27.5 |
| 65-74 | 27.4 | 27.0 | 27.3 | 27.6 | 27.7 | 27.7 |  | 26.8 | 26.2 | 26.5 | 26.8 | 27.2 | 27.5 |
| ≥75 | 26.3 | 26.0 | 26.4 | 26.7 | 26.3 | 26.0 |  | 25.8 | 25.2 | 25.9 | 26.2 | 25.7 | 26.0 |

Q1 = least deprived; Q5 = most deprived. Estimates for 25+ were age-standardised using the European Union (EU) standard population.

**Table I. Optimal scenario: risk factor levels by age-group, gender and deprivation quintile**

| **Risk factor and age** |  |  | **Men** |  |  |  |  |  |  | **Women** |  |  |  |
| --- | --- | --- | --- | --- | --- | --- | --- | --- | --- | --- | --- | --- | --- |
|  | **England** | **Q1** | **Q2** | **Q3** | **Q4** | **Q5** |  | **England** | **Q1** | **Q2** | **Q3** | **Q4** | **Q5** |
| **Smoking (%)** | | | | | | | | | | | | | |
| ***≥25*** | ***10.0*** | ***6.7*** | ***8.0*** | ***9.5*** | ***11.6*** | ***15.2*** |  | ***10.0*** | ***6.4*** | ***7.6*** | ***9.9*** | ***12.1*** | ***15.3*** |
| 25-34 | 14.3 | 11.0 | 12.3 | 13.2 | 16.0 | 17.3 |  | 11.9 | 8.6 | 9.1 | 12.1 | 13.0 | 15.1 |
| 35-44 | 11.9 | 7.7 | 9.5 | 11.4 | 14.0 | 17.7 |  | 12.0 | 7.1 | 8.8 | 11.7 | 15.6 | 17.4 |
| 45-54 | 9.8 | 6.4 | 7.3 | 9.6 | 10.5 | 17.7 |  | 10.8 | 6.4 | 8.3 | 11.1 | 12.5 | 19.0 |
| 55-64 | 7.9 | 4.7 | 5.7 | 8.0 | 10.5 | 13.9 |  | 8.7 | 5.6 | 6.4 | 8.3 | 11.5 | 15.4 |
| 65-74 | 5.3 | 3.0 | 4.9 | 4.5 | 6.7 | 8.6 |  | 6.1 | 3.7 | 5.0 | 5.8 | 7.9 | 9.7 |
| ≥75 | 3.1 | 1.8 | 2.3 | 3.0 | 4.0 | 6.1 |  | 3.8 | 2.2 | 3.6 | 3.6 | 4.8 | 5.6 |
| **Diabetes (%)** | | | | | | | | | | | | | |
| ***≥25*** | ***4.7*** | ***3.2*** | ***3.3*** | ***3.9*** | ***4.1*** | ***6.1*** |  | ***3.3*** | ***3.6*** | ***2.8*** | ***3.5*** | ***4.7*** | ***6.5*** |
| 25-34 | 0.6 | 0.2 | 0.8 | 0.4 | 0.3 | 1.0 |  | 0.9 | 2.3 | 0.8 | 0.8 | 1.0 | 0.8 |
| 35-44 | 2.3 | 1.9 | 1.7 | 0.8 | 2.6 | 2.6 |  | 1.2 | 1.0 | 1.1 | 1.8 | 0.8 | 2.8 |
| 45-54 | 4.1 | 1.9 | 2.4 | 4.4 | 3.0 | 6.7 |  | 2.7 | 2.4 | 1.8 | 3.0 | 3.5 | 6.7 |
| 55-64 | 7.2 | 6.3 | 3.7 | 6.0 | 6.3 | 9.9 |  | 4.7 | 5.4 | 4.1 | 4.5 | 7.1 | 9.4 |
| 65-74 | 11.9 | 7.8 | 10.0 | 8.4 | 11.8 | 15.0 |  | 8.2 | 7.7 | 7.1 | 8.1 | 16.3 | 13.5 |
| ≥75 | 10.1 | 6.4 | 8.8 | 11.8 | 7.3 | 8.6 |  | 8.6 | 8.9 | 8.7 | 9.6 | 9.0 | 18.7 |
| **Physical inactivity (%)** | | | | | | | | | | | | | |
| ***≥25*** | ***30.0*** | ***29.1*** | ***29.3*** | ***29.7*** | ***30.1*** | ***32.3*** |  | ***30.0*** | ***29.6*** | ***29.3*** | ***29.7*** | ***30.2*** | ***31.9*** |
| 25-34 | 25.7 | 25.5 | 25.5 | 25.3 | 24.8 | 27.1 |  | 27.5 | 29.1 | 26.9 | 27.8 | 26.5 | 27.6 |
| 35-44 | 28.3 | 26.6 | 27.4 | 28.3 | 28.1 | 31.4 |  | 28.3 | 28.1 | 27.4 | 28.1 | 28.6 | 29.3 |
| 45-54 | 30.6 | 29.1 | 29.1 | 31.4 | 31.4 | 32.7 |  | 29.6 | 27.5 | 28.1 | 29.9 | 30.8 | 33.5 |
| 55-64 | 32.0 | 30.9 | 31.2 | 32.4 | 31.4 | 35.0 |  | 30.6 | 29.7 | 30.7 | 29.2 | 30.8 | 34.1 |
| 65-74 | 33.5 | 33.6 | 33.8 | 30.1 | 34.6 | 36.2 |  | 33.6 | 33.6 | 32.8 | 32.7 | 34.5 | 35.2 |
| ≥75 | 37.5 | 37.3 | 36.9 | 35.8 | 39.9 | 38.5 |  | 38.0 | 37.5 | 37.9 | 38.0 | 37.9 | 38.7 |
| **Systolic blood pressure (mm Hg)** | | | | | | | | | | | | | |
| ***≥25*** | ***120.8*** | ***120.2*** | ***120.9*** | ***121.0*** | ***121.0*** | ***121.2*** |  | ***114.0*** | ***113.1*** | ***113.7*** | ***113.9*** | ***114.7*** | ***115.0*** |
| 25-34 | 116.4 | 116.0 | 118.2 | 116.1 | 115.9 | 116.2 |  | 103.9 | 103.6 | 104.3 | 103.9 | 104.4 | 103.3 |
| 35-44 | 117.3 | 117.0 | 117.3 | 118.1 | 117.7 | 116.4 |  | 107.0 | 106.6 | 106.7 | 106.9 | 107.4 | 107.7 |
| 45-54 | 120.3 | 119.7 | 119.9 | 120.6 | 120.5 | 121.6 |  | 113.8 | 112.2 | 113.2 | 113.7 | 115.4 | 115.4 |
| 55-64 | 124.5 | 123.7 | 124.3 | 124.6 | 125.0 | 125.6 |  | 120.7 | 119.0 | 119.5 | 121.5 | 122.0 | 123.0 |
| 65-74 | 126.8 | 125.7 | 126.3 | 128.0 | 127.9 | 126.8 |  | 127.7 | 127.2 | 128.9 | 126.8 | 126.4 | 129.8 |
| ≥75 | 128.5 | 128.2 | 127.9 | 127.8 | 128.5 | 131.7 |  | 131.6 | 132.0 | 130.6 | 130.6 | 132.8 | 132.7 |
| **Total cholesterol (mmol/l)** | | | | | | | | | | | | | |
| ***≥25*** | ***4.9*** | ***4.9*** | ***4.9*** | ***4.9*** | ***4.8*** | ***4.8*** |  | ***4.9*** | ***4.9*** | ***4.9*** | ***4.9*** | ***4.9*** | ***4.9*** |
| 25-34 | 4.6 | 4.6 | 4.6 | 4.5 | 4.6 | 4.6 |  | 4.3 | 4.3 | 4.3 | 4.3 | 4.2 | 4.3 |
| 35-44 | 5.1 | 5.1 | 5.1 | 5.1 | 5.1 | 4.9 |  | 4.6 | 4.6 | 4.6 | 4.5 | 4.7 | 4.7 |
| 45-54 | 5.2 | 5.2 | 5.3 | 5.2 | 5.1 | 5.1 |  | 5.1 | 5.1 | 5.1 | 5.1 | 5.1 | 5.2 |
| 55-64 | 5.0 | 5.1 | 5.1 | 5.1 | 5.0 | 4.8 |  | 5.5 | 5.5 | 5.6 | 5.5 | 5.3 | 5.4 |
| 65-74 | 4.6 | 4.7 | 4.6 | 4.7 | 4.5 | 4.4 |  | 5.3 | 5.3 | 5.3 | 5.3 | 5.2 | 5.2 |
| ≥75 | 4.3 | 4.3 | 4.3 | 4.3 | 4.1 | 4.3 |  | 5.0 | 5.1 | 5.1 | 5.1 | 5.0 | 4.9 |
| **Body mass index (kg/m^2^)** | | | | | | | | | | | | | |
| ***≥25*** | ***25.9*** | ***25.7*** | ***26.0*** | ***26.0*** | ***26.1*** | ***25.8*** |  | ***24.1*** | ***23.5*** | ***23.7*** | ***24.1*** | ***24.4*** | ***24.9*** |
| 25-34 | 24.7 | 24.9 | 25.1 | 24.6 | 24.7 | 24.5 |  | 22.9 | 22.7 | 22.5 | 22.7 | 23.1 | 23.5 |
| 35-44 | 26.0 | 25.7 | 26.2 | 26.0 | 26.3 | 25.7 |  | 23.9 | 23.1 | 23.4 | 23.8 | 24.3 | 24.9 |
| 45-54 | 26.3 | 26.2 | 26.3 | 26.4 | 26.6 | 26.1 |  | 24.5 | 23.8 | 24.0 | 24.5 | 25.0 | 25.5 |
| 55-64 | 26.5 | 26.2 | 26.6 | 26.7 | 26.6 | 26.7 |  | 24.8 | 24.1 | 24.5 | 24.9 | 25.2 | 25.8 |
| 65-74 | 26.4 | 26.0 | 26.3 | 26.6 | 26.7 | 26.7 |  | 25.1 | 24.6 | 24.8 | 25.1 | 25.5 | 25.8 |
| ≥75 | 25.4 | 25.1 | 25.5 | 25.7 | 25.4 | 25.1 |  | 24.2 | 23.7 | 24.2 | 24.6 | 24.1 | 24.4 |

Q1 = least deprived; Q5 = most deprived. Estimates for 25+ were age-standardised using EU standard population.

**Table J. Population in 2020, baseline mortality rates, and expected deaths assuming no change in CHD mortality rates by age-group, gender and deprivation quintile**

| **Age** |  |  | **Men** |  |  |  |  |  |  | **Women** |  |  |  |
| --- | --- | --- | --- | --- | --- | --- | --- | --- | --- | --- | --- | --- | --- |
|  | **England** | **Q1** | **Q2** | **Q3** | **Q4** | **Q5** |  | **England** | **Q1** | **Q2** | **Q3** | **Q4** | **Q5** |
| **Population in 2020 (1000s)** | | | | | | | | | | | | | |
| ***≥25*** | ***19462*** | ***4209*** | ***4130*** | ***4001*** | ***3753*** | ***3369*** |  | ***20325*** | ***4442*** | ***4345*** | ***4180*** | ***3881*** | ***3477*** |
| 25-34 | 4108 | 616 | 688 | 827 | 988 | 989 |  | 3947 | 594 | 657 | 785 | 939 | 972 |
| 35-44 | 3601 | 717 | 701 | 719 | 749 | 715 |  | 3497 | 729 | 697 | 692 | 701 | 679 |
| 45-54 | 3627 | 846 | 788 | 735 | 663 | 596 |  | 3687 | 862 | 807 | 750 | 670 | 598 |
| 55-64 | 3296 | 843 | 806 | 698 | 537 | 412 |  | 3448 | 893 | 846 | 729 | 558 | 421 |
| 65-74 | 2586 | 639 | 618 | 547 | 430 | 351 |  | 2831 | 698 | 668 | 594 | 480 | 391 |
| 75-84 | 1616 | 402 | 381 | 339 | 274 | 219 |  | 1925 | 451 | 442 | 410 | 346 | 276 |
| ≥85 | 628 | 147 | 148 | 136 | 111 | 86 |  | 992 | 215 | 229 | 219 | 188 | 140 |
| **CHD rate in 2007 (per 100,000)^a^** | | | | | | | | | | | | | |
| ***≥25*** | ***200.3*** | ***148.1*** | ***169.5*** | ***192.6*** | ***230.2*** | ***292.6*** |  | ***94.6*** | ***69.7*** | ***80.8*** | ***91.0*** | ***108.0*** | ***135.5*** |
| 25-34 | 2.5 | 1.9 | 1.8 | 1.8 | 2.4 | 4.4 |  | 0.6 | 0.3 | 0.5 | 0.5 | 0.7 | 0.8 |
| 35-44 | 16.3 | 8.6 | 10.2 | 12.8 | 20.3 | 30.1 |  | 3.7 | 1.3 | 1.7 | 3.1 | 4.5 | 8.1 |
| 45-54 | 65.8 | 35.3 | 43.4 | 57.4 | 82.3 | 123.6 |  | 13.3 | 6.2 | 8.8 | 10.6 | 15.3 | 29.3 |
| 55-64 | 178.0 | 104.0 | 132.9 | 162.6 | 218.1 | 334.4 |  | 45.2 | 20.4 | 29.7 | 40.1 | 61.1 | 96.9 |
| 65-74 | 454.8 | 303.1 | 362.2 | 433.7 | 536.7 | 734.0 |  | 180.9 | 106.7 | 133.0 | 167.0 | 220.3 | 320.5 |
| 75-84 | 1230.8 | 972.4 | 1110.0 | 1215.1 | 1402.2 | 1593.9 |  | 669.5 | 500.6 | 597.0 | 662.6 | 750.1 | 888.4 |
| ≥85 | 2802.6 | 2654.3 | 2743.1 | 2847.1 | 2905.6 | 2915.0 |  | 2033.0 | 1878.3 | 1967.7 | 2028.4 | 2161.8 | 2161.2 |
| **Expected deaths in 2020^b^** | | | | | | | | | | | | | |
| ***≥25*** | ***57270*** | ***10998*** | ***12021*** | ***12021*** | ***11283*** | ***10946*** |  | ***39789*** | ***7287*** | ***8361*** | ***8557*** | ***8200*** | ***7384*** |
| 25-34 | 106 | 12 | 12 | 15 | 23 | 43 |  | 22 | 1 | 3 | 4 | 6 | 8 |
| 35-44 | 592 | 62 | 72 | 92 | 152 | 215 |  | 130 | 10 | 12 | 21 | 31 | 55 |
| 45-54 | 2345 | 299 | 342 | 422 | 545 | 736 |  | 481 | 53 | 71 | 79 | 102 | 175 |
| 55-64 | 5632 | 876 | 1070 | 1135 | 1172 | 1379 |  | 1475 | 182 | 251 | 292 | 341 | 408 |
| 65-74 | 11437 | 1936 | 2240 | 2373 | 2310 | 2579 |  | 4935 | 745 | 889 | 992 | 1057 | 1252 |
| 75-84 | 19598 | 3905 | 4235 | 4124 | 3848 | 3487 |  | 12659 | 2256 | 2637 | 2717 | 2598 | 2451 |
| ≥85 | 17560 | 3908 | 4050 | 3861 | 3233 | 2507 |  | 20087 | 4038 | 4499 | 4452 | 4065 | 3034 |

Q1 = least deprived; Q5 = most deprived.

^a^ Three-year moving averages based on 2006-2008 data. Rates for 25+ were age-standardised using the EU standard population. CHD mortality rate for England is a direct estimate (i.e., ***not*** an average of the IMD rates).

**^b^** Expected deaths in England obtained by summation of the IMD counts.

**Table K. Deaths prevented/postponed in each scenario by age-group, gender and deprivation quintile**

| **Scenario and age** | **Men** | | | | | |  | **Women** | | | | | |
| --- | --- | --- | --- | --- | --- | --- | --- | --- | --- | --- | --- | --- | --- |
|  | **England** | **Q1** | **Q2** | **Q3** | **Q4** | **Q5** |  | **England** | **Q1** | **Q2** | **Q3** | **Q4** | **Q5** |
|  | |  |  |  |  |  |  |  |  |  |  |  |  |
| ***Worst case*** |  |  |  |  |  |  |  |  |  |  |  |  |  |
| ***≥25*** | ***8700*** | ***1410*** | ***1770*** | ***2010*** | ***1650*** | ***1860*** |  | ***7470*** | ***1260*** | ***1420*** | ***1530*** | ***1510*** | ***1750*** |
| 25-34 | 10 | 0 | 0 | 0 | 0 | 10 |  | 0 | 0 | 0 | 0 | 0 | 0 |
| 35-44 | 90 | 10 | 10 | 10 | 30 | 40 |  | 20 | 0 | 0 | 0 | 0 | 10 |
| 45-54 | 340 | 30 | 40 | 60 | 70 | 140 |  | 90 | 10 | 10 | 10 | 20 | 40 |
| 55-64 | 970 | 150 | 140 | 190 | 200 | 300 |  | 290 | 30 | 40 | 50 | 70 | 100 |
| 65-74 | 2010 | 290 | 380 | 370 | 430 | 530 |  | 1010 | 130 | 150 | 180 | 260 | 290 |
| 75-84 | 2800 | 470 | 620 | 710 | 500 | 500 |  | 2380 | 390 | 450 | 490 | 460 | 590 |
| ≥85 | 2470 | 460 | 580 | 660 | 420 | 350 |  | 3680 | 690 | 760 | 800 | 700 | 720 |
| ***Assuming current trends continue*** | | | | | | | | | | | | | |
| ***≥25*** | ***-12820*** | ***-2660*** | ***-2700*** | ***-2590*** | ***-2510*** | ***-2350*** |  | ***-9820*** | ***-1710*** | ***-2300*** | ***-1740*** | ***-2100*** | ***-1970*** |
| 25-34 | -20 | -0 | 0 | 0 | -10 | 0 |  | -10 | 0 | 0 | 0 | 0 | 0 |
| 35-44 | -100 | 10 | 0 | -20 | -10 | -70 |  | -30 | 0 | 0 | -10 | 0 | 10 |
| 45-54 | -430 | -60 | -30 | 40 | -170 | -200 |  | -30 | -10 | -20 | -10 | 10 | -10 |
| 55-64 | -1440 | -150 | -330 | -350 | -330 | -270 |  | -300 | -30 | -50 | -120 | -40 | -60 |
| 65-74 | -3240 | -430 | -560 | -550 | -630 | -1080 |  | -1360 | -130 | -370 | -370 | -190 | -310 |
| 75-84 | -4570 | -1150 | -1040 | -1020 | -840 | -520 |  | -4240 | -750 | -920 | -680 | -980 | -910 |
| ≥85 | -3030 | -880 | -730 | -690 | -530 | -210 |  | -3840 | -780 | -930 | -540 | -910 | -680 |
| ***Intermediate*** | | | | | | | | | | | | | |
| ***≥25*** | ***-13140*** | ***-2340*** | ***-2640*** | ***-2710*** | ***-2660*** | ***-2800*** |  | ***-9190*** | ***-1590*** | ***-1860*** | ***-1940*** | ***-1920*** | ***-1890*** |
| 25-34 | -50 | -10 | -10 | -10 | -10 | -20 |  | -10 | 0 | 0 | 0 | 0 | 0 |
| 35-44 | -260 | -30 | -30 | -40 | -70 | -90 |  | -60 | 0 | -10 | -10 | -20 | -30 |
| 45-54 | -1010 | -130 | -150 | -180 | -230 | -320 |  | -230 | -20 | -30 | -40 | -50 | -80 |
| 55-64 | -2010 | -300 | -370 | -410 | -420 | -510 |  | -560 | -70 | -90 | -110 | -130 | -160 |
| 65-74 | -3150 | -520 | -610 | -640 | -650 | -740 |  | -1560 | -230 | -280 | -310 | -340 | -410 |
| 75-84 | -3920 | -760 | -840 | -830 | -770 | -720 |  | -3150 | -550 | -650 | -670 | -650 | -630 |
| ≥85 | -2740 | -600 | -630 | -600 | -500 | -400 |  | -3620 | -710 | -800 | -800 | -740 | -570 |
| ***Optimal*** |  |  |  |  |  |  |  |  |  |  |  |  |  |
| ***≥25*** | ***-23340*** | ***-4210*** | ***-4720*** | ***-4820*** | ***-4700*** | ***-4890*** |  | ***-16380*** | ***-2860*** | ***-3340*** | ***-3460*** | ***-3420*** | ***-3310*** |
| 25-34 | -70 | -10 | -10 | -10 | -20 | -30 |  | -20 | 0 | 0 | 0 | 0 | -10 |
| 35-44 | -410 | -40 | -50 | -60 | -100 | -150 |  | -100 | -10 | -10 | -20 | -20 | -40 |
| 45-54 | -1600 | -200 | -230 | -290 | -370 | -510 |  | -350 | -40 | -50 | -60 | -70 | -130 |
| 55-64 | -3360 | -510 | -630 | -680 | -710 | -850 |  | -920 | -110 | -150 | -180 | -210 | -260 |
| 65-74 | -5530 | -910 | -1080 | -1130 | -1130 | -1290 |  | -2680 | -390 | -470 | -530 | -580 | -700 |
| 75-84 | -7220 | -1410 | -1540 | -1520 | -1420 | -1320 |  | -5620 | -980 | -1160 | -1200 | -1160 | -1120 |
| ≥85 | -5140 | -1130 | -1180 | -1130 | -950 | -750 |  | -6710 | -1320 | -1490 | -1480 | -1360 | -1060 |

DPP counts rounded to the nearest 10. Positive DPPs represent additional deaths caused by adverse trends; negative DPPs represent deaths postponed through risk factor reduction. DPPs for England obtained by summation of the IMD counts.

**Table L. Deaths prevented/postponed with 95% uncertainty intervals in each scenario by gender and deprivation quintile**

| **Scenario** | **All** | | | | | |
| --- | --- | --- | --- | --- | --- | --- |
|  | **England** | **Q1** | **Q2** | **Q3** | **Q4** | **Q5** |
|  | |  |  |  |  |  |
| ***Worst case*** | | | | | | |
| *DPPs* | ***16,170*** | ***2670*** | ***3190*** | ***3540*** | ***3160*** | ***3610*** |
| Lower | 18,420 | 3210 | 3710 | 4090 | 3750 | 4200 |
| Upper | 13,880 | 2120 | 2560 | 2900 | 2530 | 3040 |
| ***Assuming current trends continue*** | | | | | | |
| *DPPs* | ***-22,640*** | ***-4370*** | ***-5000*** | ***-4330*** | ***-4620*** | ***-4320*** |
| Lower | -20,390 | -3630 | -4130 | -3480 | -3700 | -3400 |
| Upper | -24,980 | -5070 | -5840 | -5170 | -5470 | -5220 |
| ***Intermediate*** | | | | | | |
| *DPPs* | ***-22,330*** | ***-3930*** | ***-4490*** | ***-4640*** | ***-4580*** | ***-4680*** |
| Lower | -19,850 | -3080 | -3500 | -3630 | -3580 | -3650 |
| Upper | -24,300 | -4650 | -5410 | -5510 | -5530 | -5570 |
| ***Optimal*** | | | | | | |
| *DPPs* | ***-39,720*** | ***-7070*** | ***-8050*** | ***-8280*** | ***-8120*** | ***-8200*** |
| Lower | -37,120 | -6300 | -7190 | -7420 | -7190 | -7260 |
| Upper | -41,900 | -7780 | -8840 | -9020 | -8970 | -9040 |

| **Scenario** | **Men** | | | | | |  | **Women** | | | | | |
| --- | --- | --- | --- | --- | --- | --- | --- | --- | --- | --- | --- | --- | --- |
|  | **England** | **Q1** | **Q2** | **Q3** | **Q4** | **Q5** |  | **England** | **Q1** | **Q2** | **Q3** | **Q4** | **Q5** |
|  | |  |  |  |  |  |  |  |  |  |  |  |  |
| ***Worst-case*** |  |  |  |  |  |  |  |  |  |  |  |  |  |
| *DPPs* | ***8700*** | ***1410*** | ***1770*** | ***2010*** | ***1650*** | ***1860*** |  | ***7470*** | ***1260*** | ***1420*** | ***1530*** | ***1510*** | ***1750*** |
| Lower | 10,030 | 1810 | 2170 | 2430 | 2100 | 2310 |  | 9270 | 1650 | 1790 | 1920 | 1890 | 2130 |
| Upper | 7190 | 1000 | 1320 | 1570 | 1220 | 1380 |  | 5800 | 850 | 1020 | 1130 | 1100 | 1370 |
| ***Assuming current trends continue*** | | | | | | | | | | | | | |
| *DPPs* | ***-12,820*** | ***-2660*** | ***-2700*** | ***-2590*** | ***-2510*** | ***-2350*** |  | ***-9820*** | ***-1710*** | ***-2300*** | ***-1740*** | ***-2100*** | ***-1970*** |
| Lower | -11,140 | -2090 | -2040 | -1920 | -1800 | -1680 |  | -8230 | -1200 | -1720 | -1190 | -1480 | -1370 |
| Upper | -14,480 | -3220 | -3330 | -3200 | -3220 | -3060 |  | -11,360 | -2170 | -2860 | -2270 | -2700 | -2540 |
| ***Intermediate*** | | | | | | | | | | | | | |
| *DPPs* | ***-13140*** | ***-2340*** | ***-2640*** | ***-2710*** | ***-2660*** | ***-2800*** |  | ***-9190*** | ***-1590*** | ***-1860*** | ***-1940*** | ***-1920*** | ***-1890*** |
| Lower | -11220 | -1730 | -1860 | -1950 | -1850 | -1990 |  | -7580 | -1040 | -1250 | -1280 | -1250 | -1210 |
| Upper | -14660 | -2860 | -3310 | -3360 | -3370 | -3500 |  | -10,490 | -2120 | -2460 | -2530 | -2520 | -2480 |
| ***Optimal*** |  |  |  |  |  |  |  |  |  |  |  |  |  |
| *DPPs* | ***-23,340*** | ***-4210*** | ***-4720*** | ***-4820*** | ***-4700*** | ***-4890*** |  | ***-16,380*** | ***-2860*** | ***-3340*** | ***-3460*** | ***-3420*** | ***-3310*** |
| Lower | -21,390 | -3640 | -4070 | -4160 | -4050 | -4160 |  | -14,740 | -2350 | -2800 | -2870 | -2800 | -2720 |
| Upper | -25,100 | -4760 | -5350 | -5400 | -5340 | -5560 |  | -17,820 | -3340 | -3920 | -4020 | -4010 | -3830 |

95% uncertainty intervals (based on the 2.5^th^ and 97.5^th^ percentiles of results generated from 1000 iterations of the model) computed using Monte Carlo simulation through the Excel add-in Ersatz software (http://www.epigear.com). DPP counts rounded to the nearest 10. Positive DPPs represent additional deaths caused by adverse trends; negative DPPs represent deaths postponed through risk factor reduction. For further details on the methodology (including the chosen distributions for the input parameters and worked examples) see the supporting information for the IMPACT_SEC_ model available at (<http://www.plosmedicine.org/article/info%3Adoi%2F10.1371%2Fjournal.pmed.1001237>).

**Table M. Expected CHD mortality rates per 100,000 in each scenario by age-group, gender and deprivation quintile**

| **Scenario and age** |  |  | **Men** |  |  |  |  |  | **Women** |  |  |
| --- | --- | --- | --- | --- | --- | --- | --- | --- | --- | --- | --- |
|  | **Q1** | **Q2** | **Q3** | **Q4** | **Q5** |  | **Q1** | **Q2** | **Q3** | **Q4** | **Q5** |
| ***Baseline (2007)*** | | | | | | | | | | | |
| ***≥25*** | ***148.1*** | ***169.5*** | ***192.6*** | ***230.2*** | ***292.6*** |  | ***69.7*** | ***80.8*** | ***91.0*** | ***108.0*** | ***135.5*** |
| 25-34 | 1.9 | 1.8 | 1.8 | 2.4 | 4.4 |  | 0.3 | 0.5 | 0.4 | 0.7 | 0.8 |
| 35-44 | 8.6 | 10.2 | 12.8 | 20.3 | 30.1 |  | 1.3 | 1.7 | 3.1 | 4.5 | 8.1 |
| 45-54 | 35.3 | 43.4 | 57.4 | 82.3 | 123.6 |  | 6.2 | 8.8 | 10.6 | 15.3 | 29.3 |
| 55-64 | 104.0 | 132.9 | 162.6 | 218.1 | 334.4 |  | 20.4 | 29.7 | 40.0 | 61.1 | 96.9 |
| 65-74 | 303.1 | 362.2 | 433.7 | 536.7 | 734.0 |  | 106.7 | 133.0 | 167.0 | 220.3 | 320.4 |
| 75-84 | 972.4 | 1110.0 | 1215.1 | 1402.2 | 1593.9 |  | 500.6 | 597.0 | 662.6 | 750.1 | 888.4 |
| *≥*85 | 2654.3 | 2743.1 | 2847.1 | 2905.6 | 2915.0 |  | 1878.3 | 1967.7 | 2028.4 | 2161.7 | 2161.2 |
| ***Worst-case*** | | | | | | | | | | | |
| ***≥25*** | ***167.6*** | ***194.4*** | ***224.4*** | ***265.2*** | ***344.9*** |  | ***81.7*** | ***94.4*** | ***107.3*** | ***128.8*** | ***167.4*** |
| 25-34 | 2.1 | 2.0 | 2.0 | 2.6 | 5.0 |  | 0.3 | 0.5 | 0.5 | 0.8 | 1.0 |
| 35-44 | 10.0 | 11.7 | 14.2 | 23.8 | 35.4 |  | 1.5 | 2.0 | 3.7 | 5.1 | 10.0 |
| 45-54 | 39.0 | 48.5 | 66.2 | 92.8 | 146.5 |  | 7.1 | 9.9 | 12.3 | 18.1 | 36.2 |
| 55-64 | 121.7 | 149.9 | 189.7 | 255.4 | 406.0 |  | 24.1 | 34.4 | 46.7 | 73.9 | 119.6 |
| 65-74 | 348.7 | 424.4 | 501.8 | 636.7 | 885.7 |  | 125.5 | 155.6 | 197.4 | 273.8 | 393.7 |
| 75-84 | 1089.0 | 1272.0 | 1425.2 | 1586.0 | 1822.8 |  | 587.5 | 699.6 | 782.7 | 881.6 | 1102.5 |
| *≥*85 | 2965.7 | 3136.4 | 3332.3 | 3278.8 | 3326.3 |  | 2199.8 | 2300.9 | 2390.9 | 2535.3 | 2677.0 |
| ***Assuming current trends continue*** | | | | | | | | | | | |
| ***≥25*** | ***113.5*** | ***131.0*** | ***151.3*** | ***175.9*** | ***223.1*** |  | ***53.1*** | ***56.8*** | ***69.1*** | ***82.0*** | ***101.2*** |
| 25-34 | 1.7 | 1.6 | 1.3 | 1.8 | 4.1 |  | 0.2 | 0.3 | 0.4 | 0.5 | 0.5 |
| 35-44 | 9.4 | 10.1 | 9.6 | 19.3 | 19.8 |  | 0.9 | 1.4 | 2.1 | 4.3 | 6.1 |
| 45-54 | 28.4 | 39.2 | 62.2 | 57.0 | 89.6 |  | 4.6 | 6.1 | 9.0 | 16.6 | 31.0 |
| 55-64 | 86.1 | 91.8 | 112.6 | 155.9 | 268.0 |  | 17.1 | 23.2 | 23.6 | 53.9 | 82.5 |
| 65-74 | 235.8 | 271.3 | 334.0 | 390.0 | 428.0 |  | 88.0 | 77.6 | 104.8 | 181.1 | 242.1 |
| 75-84 | 685.7 | 838.3 | 914.8 | 1095.4 | 1357.9 |  | 334.4 | 388.3 | 495.9 | 468.4 | 557.4 |
| *≥*85 | 2058.4 | 2245.3 | 2341.3 | 2433.0 | 2673.1 |  | 1513.3 | 1561.2 | 1780.8 | 1679.7 | 1677.1 |
| ***Intermediate*** | | | | | | | | | | | |
| ***≥25*** | ***113.5*** | ***128.5*** | ***144.6*** | ***170.1*** | ***210.8*** |  | ***53.2*** | ***60.9*** | ***67.9*** | ***79.2*** | ***96.4*** |
| 25-34 | 1.1 | 1.0 | 1.0 | 1.3 | 2.5 |  | 0.1 | 0.2 | 0.2 | 0.3 | 0.4 |
| 35-44 | 4.8 | 5.7 | 7.2 | 11.3 | 17.1 |  | 0.7 | 0.9 | 1.6 | 2.3 | 4.1 |
| 45-54 | 20.4 | 24.9 | 32.6 | 46.9 | 69.5 |  | 3.4 | 4.7 | 5.6 | 8.1 | 15.4 |
| 55-64 | 68.0 | 86.5 | 104.4 | 139.3 | 211.5 |  | 12.8 | 18.6 | 24.9 | 37.7 | 59.0 |
| 65-74 | 222.4 | 263.2 | 316.6 | 386.7 | 524.5 |  | 74.1 | 91.8 | 115.0 | 149.3 | 215.3 |
| 75-84 | 782.2 | 890.1 | 971.9 | 1120.5 | 1263.8 |  | 378.6 | 450.2 | 499.0 | 562.8 | 660.5 |
| *≥*85 | 2247.1 | 2317.2 | 2401.1 | 2452.2 | 2451.2 |  | 1547.6 | 1617.8 | 1665.7 | 1770.5 | 1752.9 |
| ***Optimal*** |  |  |  |  |  |  |  |  |  |  |  |
| ***≥25*** | ***86.9*** | ***97.3*** | ***108.5*** | ***125.5*** | ***151.5*** |  | ***40.3*** | ***45.7*** | ***50.5*** | ***57.9*** | ***68.4*** |
| 25-34 | 0.6 | 0.6 | 0.6 | 0.8 | 1.4 |  | 0.1 | 0.1 | 0.1 | 0.2 | 0.2 |
| 35-44 | 2.6 | 3.1 | 3.9 | 6.3 | 9.7 |  | 0.4 | 0.5 | 0.8 | 1.2 | 2.1 |
| 45-54 | 11.6 | 14.0 | 18.1 | 26.1 | 38.2 |  | 1.8 | 2.5 | 3.0 | 4.3 | 8.2 |
| 55-64 | 43.7 | 55.2 | 65.4 | 86.6 | 129.4 |  | 7.9 | 11.4 | 15.2 | 22.6 | 35.0 |
| 65-74 | 160.6 | 188.1 | 227.8 | 273.6 | 367.3 |  | 50.5 | 62.1 | 77.6 | 99.1 | 141.4 |
| 75-84 | 621.5 | 705.3 | 768.4 | 883.4 | 989.1 |  | 282.2 | 334.6 | 370.3 | 416.1 | 483.8 |
| *≥*85 | 1880.0 | 1942.9 | 2010.4 | 2052.3 | 2045.0 |  | 1263.1 | 1317.4 | 1354.7 | 1436.0 | 1407.3 |

Rates for 25+ were age-standardised using the EU standard population. In each scenario, rate differences (RD) between the most and least deprived quintiles calculated as (Rate_Q5_-Rate_Q1_); rate ratios (RR) as (Rate_Q5_/Rate_Q1_).

Percent change in RD can be calculated as^17^: *[(RD_Q5-Q1,baseline_)–(RD_Q5-Q1,scenario_)]/[(RD_Q5-Q1,baseline_)] × 100*

Percent change in rate ratio as^17^: *[(rate ratio_Q5-Q1,baseline_)–(rate ratio_Q5-Q1,scenario_)]/[(rate ratio_Q5-Q1,baseline_)-1] × 100*

**Table N. Relative change in CHD mortality (%) in each scenario by age-group, gender and deprivation quintile**

| **Scenario and age** |  |  | **Men** |  |  |  |  |  | **Women** |  |  |
| --- | --- | --- | --- | --- | --- | --- | --- | --- | --- | --- | --- |
|  | **Q1** | **Q2** | **Q3** | **Q4** | **Q5** |  | **Q1** | **Q2** | **Q3** | **Q4** | **Q5** |
| ***Worst case*** | | | | | | | | | | | |
| ***≥25*** | ***13.2*** | ***14.7*** | ***16.5*** | ***15.2*** | ***17.9*** |  | ***17.3*** | ***16.9*** | ***17.9*** | ***19.3*** | ***23.6*** |
| 25-34 | 8.7 | 12.5 | 9.5 | 8.7 | 13.7 |  | 21.0 | 13.6 | 13.2 | 15.0 | 13.9 |
| 35-44 | 15.2 | 14.4 | 10.9 | 17.7 | 17.7 |  | 13.7 | 14.3 | 18.2 | 12.9 | 22.6 |
| 45-54 | 10.4 | 11.6 | 15.4 | 12.8 | 18.5 |  | 15.1 | 12.8 | 16.7 | 18.1 | 23.8 |
| 55-64 | 17.0 | 12.8 | 16.7 | 17.1 | 21.4 |  | 18.2 | 16.0 | 16.7 | 20.9 | 23.4 |
| 65-74 | 15.0 | 17.2 | 15.7 | 18.6 | 20.7 |  | 17.6 | 17.0 | 18.2 | 24.3 | 22.8 |
| 75-84 | 12.0 | 14.6 | 17.3 | 13.1 | 14.4 |  | 17.4 | 17.2 | 18.1 | 17.5 | 24.1 |
| *≥*85 | 11.7 | 14.3 | 17.0 | 12.8 | 14.1 |  | 17.1 | 16.9 | 17.9 | 17.3 | 23.9 |
| ***Assuming current trends continue*** | | | | | | | | | | | |
| ***≥25*** | ***-23.3*** | ***-22.7*** | ***-21.4*** | ***-23.6*** | ***-23.7*** |  | ***-23.8*** | ***-29.7*** | ***-24.0*** | ***-24.1*** | ***-25.3*** |
| 25-34 | -12.0 | -11.0 | -26.6 | -25.4 | -6.4 |  | -37.0 | -24.7 | -13.4 | -22.4 | -42.3 |
| 35-44 | 9.0 | -0.6 | -25.1 | -4.9 | -34.1 |  | -36.0 | -17.9 | -32.5 | -4.8 | -24.7 |
| 45-54 | -19.6 | -9.8 | 8.4 | -30.7 | -27.5 |  | -25.3 | -30.3 | -14.5 | 8.7 | 6.0 |
| 55-64 | -17.2 | -30.9 | -30.8 | -28.5 | -19.9 |  | -16.1 | -21.8 | -41.1 | -11.8 | -14.9 |
| 65-74 | -22.2 | -25.1 | -23.0 | -27.3 | -41.7 |  | -17.5 | -41.7 | -37.2 | -17.8 | -24.5 |
| 75-84 | -29.5 | -24.5 | -24.7 | -21.9 | -14.8 |  | -33.2 | -35.0 | -25.2 | -37.6 | -37.3 |
| *≥*85 | -22.5 | -18.1 | -17.8 | -16.3 | -8.3 |  | -19.4 | -20.7 | -12.2 | -22.3 | -22.4 |
| ***Intermediate*** | | | | | | | | | | | |
| ***≥25*** | ***-23.3*** | ***-24.2*** | ***-24.9*** | ***-26.1*** | ***-28.0*** |  | ***-23.7*** | ***-24.6*** | ***-25.4*** | ***-26.7*** | ***-28.8*** |
| 25-34 | -43.5 | -43.8 | -43.3 | -42.9 | -43.2 |  | -24.1 | -19.5 | -27.0 | -29.4 | -32.4 |
| 35-44 | -44.4 | -44.2 | -44.0 | -44.1 | -43.1 |  | -58.2 | -64.3 | -69.5 | -65.8 | -54.3 |
| 45-54 | -42.2 | -42.6 | -43.2 | -43.0 | -43.7 |  | -57.1 | -67.0 | -60.3 | -72.5 | -66.0 |
| 55-64 | -34.6 | -34.9 | -35.8 | -36.1 | -36.7 |  | -61.2 | -59.0 | -67.2 | -74.4 | -73.6 |
| 65-74 | -26.6 | -27.3 | -27.0 | -27.9 | -28.5 |  | -53.7 | -50.9 | -35.7 | -58.0 | -57.5 |
| 75-84 | -19.6 | -19.8 | -20.0 | -20.1 | -20.7 |  | -42.7 | -19.9 | -26.0 | -45.3 | -41.6 |
| *≥*85 | -15.3 | -15.5 | -15.7 | -15.6 | -15.9 |  | -15.6 | -13.8 | -25.3 | -11.2 | -13.2 |
| ***Optimal*** |  |  |  |  |  |  |  |  |  |  |  |
| ***≥25*** | ***-41.3*** | ***-42.6*** | ***-43.6*** | ***-45.5*** | ***-48.2*** |  | ***-42.1*** | ***-43.4*** | ***-44.5*** | ***-46.4*** | ***-49.5*** |
| 25-34 | -68.8 | -69.0 | -68.3 | -67.7 | -67.8 |  | -73.6 | -73.1 | -73.6 | -73.5 | -73.6 |
| 35-44 | -69.8 | -69.5 | -69.2 | -69.1 | -67.7 |  | -72.5 | -72.9 | -73.2 | -73.8 | -74.4 |
| 45-54 | -67.3 | -67.7 | -68.5 | -68.2 | -69.1 |  | -71.0 | -71.4 | -72.0 | -72.2 | -72.0 |
| 55-64 | -58.0 | -58.5 | -59.8 | -60.3 | -61.3 |  | -61.2 | -61.6 | -62.1 | -63.0 | -63.9 |
| 65-74 | -47.0 | -48.1 | -47.5 | -49.0 | -50.0 |  | -52.7 | -53.3 | -53.5 | -55.0 | -55.9 |
| 75-84 | -36.1 | -36.5 | -36.8 | -37.0 | -37.9 |  | -43.6 | -44.0 | -44.1 | -44.5 | -45.5 |
| *≥*85 | -28.9 | -29.2 | -29.4 | -29.4 | -29.8 |  | -32.8 | -33.0 | -33.2 | -33.6 | -34.9 |

Relative change in CHD mortality rates for each quintile calculated as:

*(CHD mortality rate_scenario_ - CHD mortality rate_baseline_)/CHD mortality rate_baseline_*

Positive values for relative change represent increasing mortality rates; negative values represent declines in mortality rates.

**References**

1. Lewington S, Whitlock G, Clarke R, Sherliker P, Emberson J, et al. (2007) Blood cholesterol and vascular mortality by age, sex, and blood pressure: a meta-analysis of individual data from 61 prospective studies with 55000 vascular deaths. Lancet 370:1829-1839.

2. Bogers RP, Hoogenveen RT, Boshuizen H, Woodward M, Knekt P, et al. (2006) A pooled analysis of 30 prospective studies. European Journal of Epidemiology 21(Suppl):313.

3. James WPT, Jackson-Leach R, Mhurchu CN, Kalamara E, Shayeghi M, et al. Overweight and obesity (high body mass index). In: Ezzati M, Lopez AD, Rodgers A, Murray CJL, eds. *Comparative quantification of risk. Global and regional burden of disease attributable to selected major risk factors. Volume 1.* World Health Organization, 2004.

4. Lewington S, Clarke R, Qizilbash N, Peto R, Collins R. (2002) Age-specific relevance of usual blood pressure to vascular mortality: a meta-analysis of individual data for one million adults in 61 prospective studies. Lancet 360:1903-1913.

5. Kirkwood BR, Sterne JAC. Essential medical statistics 2^nd^ edition*.* Blackwell Science; 2003.

6. Bajekal M, Scholes S, Love H, Hawkins N, O’Flaherty M, et al. (2012) Analysing recent socioeconomic trends in coronary heart disease mortality in England, 2000-2007: a population modelling study. PLoS Med 9(6):e1001237.

7. Ezzati M, Lopez AD, Rodgers A, Murray CJL, eds. *Comparative quantification of risk. Global and regional burden of disease attributable to selected major risk factors. Volume 1.* World Health Organization, 2004.

8. Roglic G, Unwin N. (2010) Mortality attributable to disease: estimates for the year 2010. Diabetes Res Clin Pract 87:15-19.

9. Huxley R, Barzi F, Woodward M. (2006) Excess risk of fatal coronary heart disease associated with diabetes in men and women: meta-analysis of 37 prospective cohort studies. BMJ 332:73-78.

10. Tobias M, Taylor R, Yeh LC, Huang K, Mann S, et al. (2008) Did it fall or was it pushed? The contribution of trends in established risk factors to the decline of coronary heart disease mortality in New Zealand. Aust N Z J Public Health 32:117-125.

11. Taylor R, Dobson A, Mirzaei M. (2006) Contribution of changes in risk factors to the decline of coronary heart disease mortality in Australia over three decades. European Journal of Cardiovascular Prevention and Rehabilitation 13:760-768.

12. Yusuf S. (2002) Two decades of progress in preventing vascular disease. Lancet 360:2-3.

13. Craig R, Shelton N, eds. Health Survey for England 2007: Healthy lifestyles: knowledge, attitudes and behaviour. The Information Centre, 2008. (<http://www.ic.nhs.uk/pubs/hse07healthylifestyles>)

14. Scholes S, Bajekal M, Love H, Hawkins N, Raine R, et al. (2012) Persistent socioeconomic inequalities in cardiovascular risk factors in England over 1994-2008: a time-trend analysis of repeated cross-sectional data. BMC Public Health: 12:129.

15. Mindell J, Biddulph JP, Hirani V, Stamatakis E, Craig R, et al. (2012) Cohort profile: The Health Survey for England. Int J Epidemiol doi: 10.1093/ije/dyr199.

16. Stewart ST, Cutler DM, Rosen AB. (2009) Forecasting the effects of obesity and smoking on U.S. life expectancy. New England Journal of Medicine 361:2252-2260.

17. Alvardo BE, Harper S, Platt RW, Davey Smith G, Lynch J. (2009) Would achieving Healthy People 2010’s targets reduce both population levels and social disparities in heart disease? Circ Cardiovasc Qual Outcomes;2:598-606. [See Supplemental Methods: pp.12-13].
